# Statistical evidence for the contribution of citizen-led initiatives and projects to the energy transition in Europe

**DOI:** 10.1038/s41598-023-28504-4

**Published:** 2023-03-02

**Authors:** Valeria Jana Schwanitz, August Wierling, Heather Arghandeh Paudler, Constantin von Beck, Simon Dufner, Ingrid Knutsdotter Koren, Tobias Kraudzun, Timothy Marcroft, Lukas Mueller, Jan Pedro Zeiss

**Affiliations:** 1 Department of Environmental Sciences, Western Norway University of Applied Sciences, 113-8656 Sogndal, Norway; 2 The Schumacher Institute, The Create Centre, Bristol, BS1 6XN UK; 3 Present Address: Department of Business Administration, Economics and Law, Technical University of Darmstadt, 64289 Darmstadt, Germany

**Keywords:** Energy and society, Environmental economics, Sustainability

## Abstract

Statistical accounting of the impacts of citizen-led energy initiatives is absent, despite their impact on increased energy self-sufficiency and ramping up of renewable energies, local sustainable development, greater citizen engagement, diversification of activities, social innovation, and acceptance of transition measures. This paper quantifies the aggregate contributions of collective action in pursuit of the sustainable energy transition in Europe. We estimate the number of initiatives (10,540), projects (22,830), people involved (2,010,600), installed renewable capacities (7.2–9.9 GW), and investments made (6.2–11.3 billion EUR) for 30 European countries. Our aggregate estimates do not suggest that collective action will replace commercial enterprises and governmental action in the short or medium term without fundamental alterations to policy and market structures. However, we find strong evidence for the historical, emerging, and actual importance of citizen-led collective action to the European energy transition. Collective action in the energy transition is experimenting successfully with new business models in the energy sector. Continued decentralization of energy systems and more stringent decarbonization policies will increase the importance of these actors in the future.

## Introduction

Clean, secure, and affordable are key words in the ongoing energy transition toward a zero-carbon global energy sector^1^. Enabling and accelerating these goals requires massive mobilization of resources to lower greenhouse gas emissions and increase resource efficiency of energy and material systems^2^. Estimates predict global investment needs of $2063 billion annually between 2022 and 2025 and an average of $4189 billion per year thereafter to reach a net-zero emissions scenario by 2030^3^. While the importance of mobilizing both public and private investors across-the-board is emphasized^4^, citizen-led initiatives and their manifold contributions have been systematically overlooked. This is despite their active involvement in, and pivotal contributions to, for example, the electrification of rural areas in the early twentieth century^5^ or their leading role in enabling the shift towards wind energy in Denmark^6^. Focusing on the past twenty years, this paper quantifies the aggregate contributions of collective action and systematically identifies solutions in pursuit of the sustainable energy transition for European countries.

The secure, sustainable, and affordable provision of energy services for all is of prime public interest and is a goal in the international Sustainable Development Agenda^7^. Moreover, recent geopolitical turmoil and skyrocketing energy prices underline the importance of energy security and affordability. In Europe, the energy system is undergoing a stark transition driven by the liberalization of energy markets, the need to decarbonize energy and other sectors incentivized by climate policies (e.g., emission trading schemes, energy efficiency standards, carbon taxes, feed-in tariffs, and R&D grants)^2^, and on-going data-driven digitalization of the energy sector^8^. As a result, energy markets are changing from traditionally centralized systems to decentralized modes of energy services provision. These markets are opening up to new technologies, schemes of operation and management, and new market actors (as the emergence of the term 'prosumer' demonstrates). The transition requires fundamental changes in the governance of energy systems^9,10^. While the European framework for a unified energy market has been set^11^, countries differ widely in their formalizations and approaches to implementing EU legislation^12^.

Our central object of study is citizen-led energy initiatives and their aggregate contribution to the low carbon energy transition in Europe (c.f.^13–15^), which complements the contribution by individual citizens^16^. Energy cooperatives are a prime example, but not the only one. Table 1 lists citizen-led initiatives found across Europe, showcasing the variety of relevant (legal) forms and energy-related activities. The supplementary material lists prevailing types in each country, along with details on data availability, which differs across countries^17^. We estimate the number of initiatives, projects, people involved, installed renewable capacities, and investments made for 30 European countries.

**Table 1:** Types and examples of citizen-led energy initiatives found across Europe, showcasing the variety of relevant (legal) forms and energy-related activities.

Citizen-led energy initiative	Criteria compliance level: y—yes, p-partly/limited, n–no	Example of an initiative	Example(s) of projects from that initiative
Energy cooperatives	(y) Citizen-leadership	Energiegenossenschaft Starkenburg eG is a 1000-member cooperative, founded by 13 citizens in the city of Heppenheim in 2010	Owning and operating 7 wind and 31 solar photovoltaic projects, also provides consulting and information services
	(y) Social and/or environmental benefit		
	(y) Active in energy		
Renewable energy communities	(p) Citizen-leadership, but often initiated by municipalities	Comunità energetica di Borutta is an Italian renewable energy community (CER) without a separate legal form, operating in the town of Borutta since 2020	Installation and operation of a 850 kW wind turbine and photovoltaic roof-top systems on town hall, sport centers, and schools. Striving for free-of-charge, self-produced electricity. Another motivation is halting rural depopulation
	(y) Social and/or environmental benefit		
	(y) Active in energy		
Energy communities	(p) Citizen-leadership, but often initiated by municipalities, local authorities and sometimes seen as an opportunity for companies	Minoan Energy in Crete was established in 2019 in Greece and is registered under the legal form of a cooperative. It has 313 members (incl. three Municipalities and the Region of Crete)	Operates a 405 kW solar photovoltaic system, members can purchase shares to meet their households' energy demand. Offers non-profit advice to citizens and public authorities for energy saving measures and energy efficiency upgrade of buildings. Decided recently to financially support families affected by the pandemic and the earthquakes
	(y) Social and/or environmental benefit		
	(y) Active in energy		
Sustainable energy communities	(p) Citizen-leadership encouraged through general principles	Camross Parish Development Association located in Laois, Ireland, is not a legal form itself, but registered in the SEAI network	Drafted a community-led plan to develop Camross as a "smart village" and to reduce GHG emissions. Promotes behavioral change, energy-independence, and climate action
	(y) Social and/or environmental benefit		
	(y) Active in energy		
Housing cooperatives and associations	(y) Citizen-leadership	A multi-apartment residential building in Alytus, Lithuania, was registered in 2008 as a housing association	Installed a 14 kW geothermal heating system for the building. Other projects include renovation and improvement of energy efficiency
	(y) Social and/or environmental benefit		
	(p) Also active in energy (e.g., energy-efficiency measures, RE-based self-production of electricity) but housing is the primary focus		
Sustainable mobility cooperatives	(y) Citizen-leadership	Ecotxe is a consumer cooperative on the island of Palma, Spain, that practices co-ownership and sharing of electric vehicles among local citizens	This cooperative counts 240 members and 275 users for its fleet of 5 electric cars. Works in partnership with the local government to provide a service that is complementary to public transport
	(y) Social and/or environmental benefit		
	(y) Active in energy through facilitating sustainable mobility (e.g., electric vehicle rental, carsharing, rail transport sector)		
Energy clusters	(p) Limited citizen-leadership, often initiated by municipalities, local authorities, and companies	Zgorzelecki Klaster Rozwoju Odnawialnych Źródeł Energii has 100 members and was selected in 2019 in a national call to become one of the Polish energy clusters (not a legal form)	Installed photovoltaic farms with a combined capacity of 46 MW. Has comprehensive energy plans for the region (incl. mobility sector). Strives for connecting renewable production and regional development
	(y) Social and/or environmental benefit		
	(y) Active in energy		
Historic rural electrification cooperatives	(y) Citizen-leadership	Société Coopérative d'Intérêt Collectif Agricole de la Région de Pithiviers was founded in 1919 as a rural electrification cooperative, counting over 1300 members today	Now also a local distribution company for both electricity and gas, serving over 26,000 customers. Began recently developing wind parks, opening the capital to local citizens and their initiatives
	(y) Social and/or environmental benefit		
	(y) Active in energy		
Eco-villages	(y) Citizen-leadership	Tuggelite eco-village community is registered as a tenant owner’s association (Bostadsrättsförening) in Sweden. Starting with 16 households in 1984, it now has 50 households participating	Combines energy and resource conservation measures for electricity, heating, and water needs. Operates a central district heating system for wood pellets and 120 m^2^ of solar panels
	(y) Social and/or environmental benefit		
	(p) Also active in energy but have a general sustainable development perspective		
**Compared to companies and public sector**			
Type	Criteria compliance level: y—yes, p-partly/limited, n–no	Type	Criteria compliance level: y—yes, p-partly/limited, n–no
For-profit companies	(n) Shareholder-leadership	Public sector power companies	(p) Elected official and public functionary leadership
	(n) Economic success is priority		(p) Both—typically shared economic and social benefit
	(y) Active in energy		(y) Active in energy

Although we are aware that many conceptual and statistical issues exist and significant uncertainties remain, we support counting what has not yet been counted, thus bringing deserved attention. These issues would suggest that our estimates are conservative and could increase with: (1) more and broader effort (e.g., filling missing data and accounting for the contribution of individual prosumers), (2) statutory reporting requirements in all countries, and (3) timely reporting by initiatives (even obligatory reporting is often delayed by two or more years). Regarding our lack of accounting for the time-value of money, the direction of influence this would have on our estimate is not clear. While inflation suggests lower estimates, considering today's monetary values, technological learning acts in the opposite direction. Finally, the definition of the object of study has a substantial impact on the aggregate contributions that we arrive at; other research efforts with broader or more limiting definitions of collective citizen engagement in the energy transition will result in different figures, without however invalidating the overall picture of our results.

## Results

### Quantitative results at the European and country-levels

**Figure 1:**
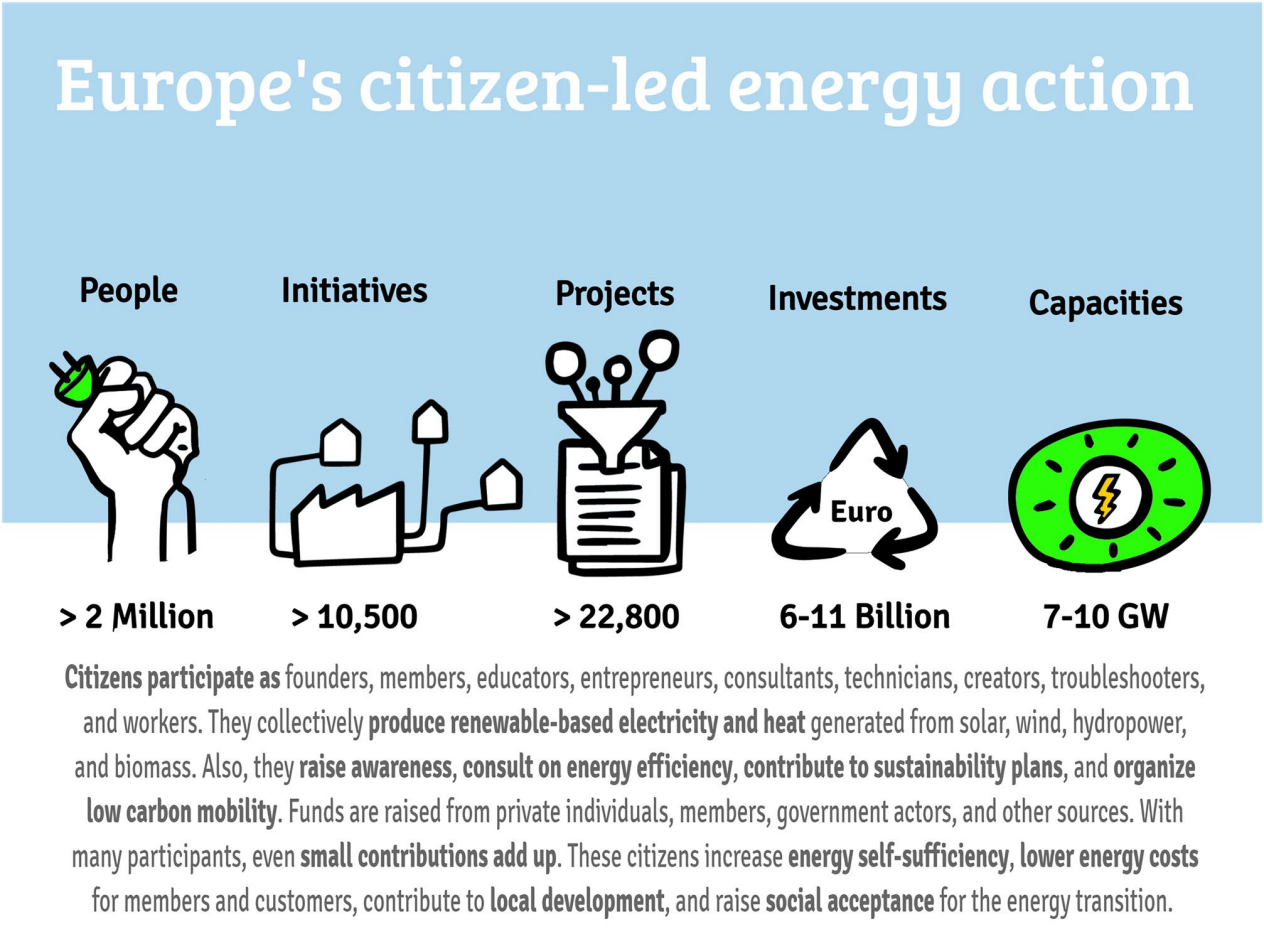
Europe-level aggregates. Contributions of citizens from 30 European countries to the energy transition. Most data collected are from 2000 to 2021.

Figure 1 shows our estimates of citizen-led contributions to the energy transition in Europe. Focusing on data from 2000 to 2021, selected estimates for the number of initiatives (10,540), people collectively engaged (2,010,600), projects undertaken (22,830), finances invested (6.2–11.3 billion EUR), and renewable capacities installed (7.2–9.9 GW) were derived from country-level aggregates of 25–30 European countries (depending on the estimate). Table 2 shows the country-level aggregates for the number of initiatives, people involved, projects, renewable capacities, and finances. Note that among the projects included are those dedicated to the production and distribution of energy (e.g., the operation, installation, and/or financing of any kind of renewable energy generation facility, distribution of electricity or heat, energy trade, collective purchasing of energy and energy-related products), the provision of energy services (e.g., low carbon self-consumption, municipal lighting contractors, car sharing and operation of EV charging stations, bike sharing, retrofitting of buildings, and energy efficiency and energy saving measures), and information & awareness actions (e.g., energy-related education and campaigns, energy consulting services). While providing aggregates for renewable capacities, we do not separately report aggregate numbers for energy saved or other activities such as in mobility or information and awareness raising as this information is highly project-specific (and hence difficult to aggregate).

**Table 2:** Selected country-level aggregates of citizen-led energy initiatives contributions in 30 European countries: number of initiatives, people involved, total number of energy projects, renewable capacities installed, and total funds invested.

Country	Number of initiatives	Number of people involved	Renewable capacities installed	Number of projects	Total funds invested
Austria	389	21,750	352 MW	430	327.7 Million EUR
Belgium	112	162,905	156–566 MW	850	199.3–690.3 Million EUR
Bulgaria	14	93	N/A	14	N/A
Croatia	15	1300	10–60 MW	16	21.94–71.94 Million EUR
Czech Republic	38	266	31 MW	42	N/A
Cyprus	2	N/A	N/A	2	N/A
Denmark	665	306,650	2613 MW	600	411–2377 Million EUR
Estonia	132	5340	13 MW	142	9.5 Million EUR
Finland	94	105,700	87–172 MW	120	N/A
France	379	130,000	139–319 MW	2010	204–455 Million EUR
Germany	5015	391,500	2157–3279 MW	11,500	3152–4614 Million EUR
Greece	192	2120	0–86 MW	240	102.621 Million EUR
Hungary	8	65	0.03 MW	8	22,500 EUR
Ireland	565	25,000	9–14 MW	565	1.8–20.3 Million EUR
Italy	207	79,420	293–348 MW	558	110.8–184.8 Million EUR
Latvia	8	150	0.1–0.13 MW	9	0.825 Million EUR
Lithuania	21	650	0.3 MW	21	4.86 Million EUR
Luxembourg	68	1200	1–25 MW	86	4.028 Million EUR
Malta	2	366	1 MW	2	0.7 Million EUR
Netherlands	999	188,400	613–1027 MW	1446	733–1282 Million EUR
Norway	36	8170	2–14 MW	36	N/A
Poland	121	71,720	142–155 MW	136	2.5 Million EUR
Portugal	37	45,000	4.4 MW	69	17.93 Million EUR
Romania	5	750	5 MW	5	0.4–4.5 Million EUR
Slovakia	25	175	15 MW	56	26.374 Million EUR
Slovenia	11	77	0.3 MW	12	0.252–0.454 Million EUR
Spain	358	185,440	101–207 MW	370	65.8–113.8 Million EUR
Sweden	336	124,500	170–265 MW	375	229.5–369.3 Million EUR
Switzerland	297	84,470	50–94 MW	2580	344.4 Million EUR
United Kingdom	387	67,425	235 MW	533	260.5 Million EUR

Most data collected are from 2000 to 2021.

In general, more detailed information is available for larger initiatives, all of which we are likely to cover with a high level of detail. Over 70% of initiatives are officially registered and over 70% have a website. Information about members and production units is available for ~ 40% and ~ 50%, respectively. Countries with the best coverage include Belgium, Denmark, Germany, and the Netherlands, whereas less information is available from the Czech Republic, Finland, Croatia, and Switzerland. When data is lacking, low (high) estimates assume 0% (100%) ownership shares of production units to calculate renewable capacities (i.e., intended full load sustained output of a facility). The high estimates also include future planned projects at their currently projected costs. Investment data are based on reported investments and add estimated investments using technology cost and capacity values when possible. For details on aggregation methods in general and for individual countries, see Supplementary Note 1.

Relating these estimates to other figures gives a clearer picture of the relative impact of these initiatives. For example, compared to the population of Europe or individual countries, the numbers of people involved in these initiatives are marginal. We observe that citizen-owned renewable capacities generally represent a small percentage of total installed renewable capacities in a given country. In the higher range, we find Belgian citizen initiatives contribute about 5% of national renewable capacities, and Danish ones contribute as much as 2.3 GW of installed district heating capacities, or roughly 75% of the country's total. Accounting for efficiency losses from the production to the consumption of electricity using a capacity factor of 27%, a rough calculation suggests that 8500–11,700 kWh are produced annually per person involved. This approximately covers the yearly electricity needs of a typical European household. That is, citizen-led energy projects have enabled renewable-based energy self-sufficiency for as many people as are engaged in the movement (households included). Note that initiatives also install renewable capacities in regions, and even countries, other than their own. Considering that the majority of investments was undertaken between 2009 and 2021, we can report that annual investments by citizen-led energy initiatives for the period ranged on average between 0.5 and 0.9 billion EUR, or about 1% of the total investments into renewable energy in Europe in that timeframe^3^. Most of these investments are in higher GDP countries, and we find an average per-member investment of 5700 EUR. Relating total investments to total initiative-installed renewable capacities, we find an investment cost of about 1.2 EUR/Watt which is within the usual order of magnitude of capital expenditure for renewable technologies. Note that due to uncertainty in ownership shares of production units, this cost figure is a lower estimate.

### The evolution of European initiatives and topics of engagement

**Figure 2:**
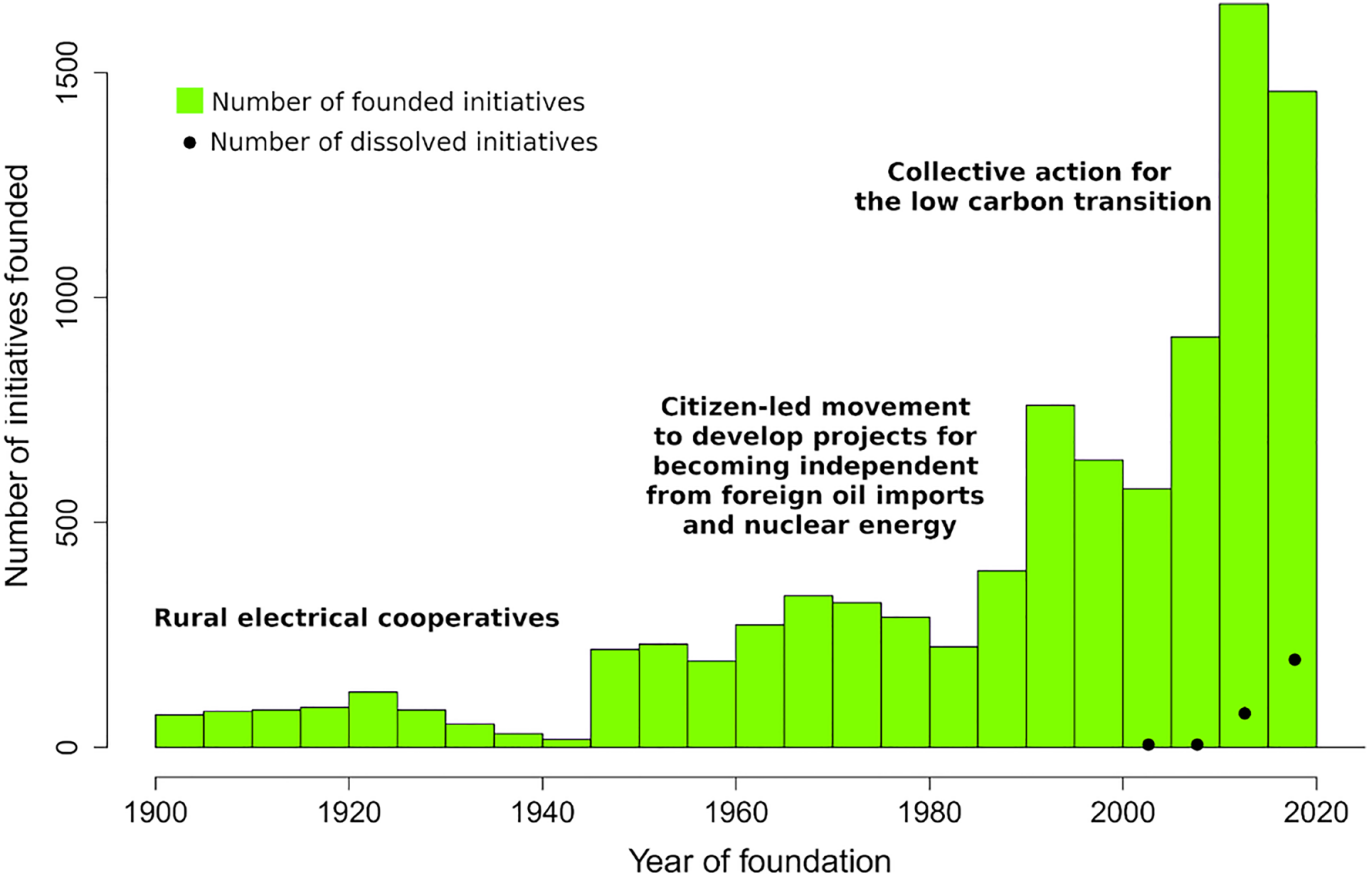
Development of initiatives 1900–2020 in Europe. Histogram with 5-year bins showing the number of newly founded and dissolved initiatives. Note that not all initiatives report the year of foundation/dissolution. Reasons for dissolution vary, including bankruptcy, merging with other organizations, or starting for-profit enterprises.

Figure 2 shows the number of newly founded, as well as dissolved, initiatives from 1900 to 2020. 89% of the initiatives in our inventory report the year of foundation, while dissolution years are seldom available, creating a bias. This suggests that the figure underestimates the number of initiatives that may have existed, and been dissolved, at some point in the past. However, we are confident in our coverage of the data available today. Figure 2 illustrates that many initiatives were founded during the past 30 years, particularly from 2010 to 2015, coinciding with the period when high feed-in tariffs were in place in many countries. These schemes were removed or lowered towards the end of this period. However, dynamics in each European country are different: while Danish initiatives strongly declined during the last decade, current trends for Croatia, Poland, Portugal, Slovenia, Italy, and Spain suggest sustained future growth. A thorough investigation of drivers and their relative importance for each country is an interesting subject for future research. It can be expected that the current ongoing implementation of EU Directives^18,19^, as well as the greater urgency of ensuring energy security and efficiency, will likely trigger the foundation of new initiatives.

The number of people involved is perhaps the most important metric when holistically considering the impact of citizen-led energy initiatives. It is not clear to which other statistics we should compare the involvement of the approximately 2 million people we observe (e.g., volunteer participation rates or measures of the maturity of civil society). According to a 2017 systematic literature review by Berka and Creamer, there is evidence and theoretical justification for members gaining new knowledge in the technical, environmental, and economic aspects of renewable energy, acquiring experience in organizing and campaigning, and becoming better informed energy consumers (and prosumers), potentially changing their behavior^20^. And yet, the figures reported here certainly underestimate the degree to which these initiatives impact general public knowledge, opinions, and actions; for every person who joins as a member, many times more will have been informed, solicited, and offered the opportunity to question their behaviors and place within the energy system. This informational halo effect, not quantified here or in the literature, could enter into considerations of the aggregate impacts of these initiatives.

Most of the 16,069 production units in the inventory are solar PV systems (82%), followed by onshore wind parks (9%), biomass-based electricity and heat production (7%), and hydropower (2%). Rarer energy production technologies include solar thermal, concentrated solar power, geothermal, and hydrogen production. While these findings reflect the fact that the former technologies are established and their kW-costs have steeply declined over the past two decades, the main driver of their adoption is that they are suitable technologies to be deployed by citizens who are volunteering part-time and may not have a background in energy. These are granular technologies, making them "more likely to scale through replication"^21^ since they are small, variable in size, modularizable, and have low risks and investment costs per unit^21^. Moreover, once installed, they are easy to operate and maintain, supporting their uptake by citizens.

Solar projects in our dataset have an average unit size of 177 kW (covering ~ 1100 m^2^). Note, however, that the median is only 29 kW (~ 200 m^2^), as the majority of units are small. Moreover, as we found and as is supported by the literature^20^, many initiatives use accumulated knowledge to sustain their activities in the energy transition, engaging in more than one project. At the same time, 68% of initiatives choose to realize just one project (representing 25% of all projects), considering their collective engagement fulfilled at project completion. Regarding wind projects, the average size is 4600 kW, with a median of 2000 kW. While Danish cooperatives were pioneers of wind parks, they have become increasingly alluring for investments by collective actions in other countries during the past decade. For example, once all current planned wind projects in the Netherlands are completed, total capacities installed by initiatives since 2000 will more than double.

Along with renewable-based electricity and heat generation, citizens also collectively engage in distribution and trade. Initiatives generating heat typically own the distribution infrastructure, while this is rare in the case of electricity production. This is partly because electricity distribution and trade comes with registration and compliance obligations regarding national grid codes, and still exists as an effective state-granted monopoly in some countries. Nevertheless, noteworthy ownership of grid infrastructure exists in Spain (16 initiatives) and in the Italian Alpine region (8 initiatives). More recently, initiatives have also invested into broadband and low-carbon mobility. For example, the number of EV charging stations installed and managed by citizen-led initiatives in Germany has been growing for the past 5 years (from 28 to 209), also in part because it provides them with an opportunity to utilize generated electricity when it is not possible to feed it into the grid.

## Discussion

The uncertainty range for our estimate of the total financial investments by citizens into collective energy projects is considerable, due to the lack of harmonized statistics and reporting obligations. For example, it is not always clear whether figures include value-added tax, creating an uncertainty range of up to 20%. It should be noted that the range for our estimate remains conservative for several reasons. First, we only include investments if evidence shows that they are energy-specific, i.e. we exclude investments into agricultural production or forestry. We also do not account for unspecified investment figures if an initiative's primary purpose is not energy focused, and we only include investments by defunct initiatives if they can be linked to a renewable production unit or other low carbon energy project. Consequently, we rely on available information of related production units. This is why we do not include grid infrastructure investments by Spanish initiatives, for example. Second, we attempt to estimate investment costs based on renewable capacities installed where possible to counterbalance the lack of investment data. This works relatively well for photovoltaic systems and wind farms, but less so for generation technologies that come with high site-specific cost. For example, in Finland where activities mainly focus on heat generation, we have a fairly small sample and lack detailed information about parameters of single production units. Thus, reliable estimates cannot be inferred, and we do not report any investment contribution from Finnish initiatives. Finally, we do not count in-kind contributions by the members. To give an idea of the orders of magnitude involved, if every member invested one hour per month, assuming minimum wages between 2 and 14 EUR/h across Europe, yearly in-kind contributions would reach roughly 227 million EUR (adding 4% to our investment estimate).

In view of the energy transition challenges ahead and recent turmoil in energy and resource markets, citizens and governments in many countries are in search of new ways to increase energy security, develop sustainable energy, and mitigate energy poverty. Our aggregate estimates do not raise expectations that collective action could replace commercial enterprises and governmental action in the short or medium term without profound changes to policy and market structures. However, we find strong evidence for the emerging and current importance of citizen-led collective action for increased energy self-sufficiency, local sustainable development, greater citizen engagement, diversification of fields of activities, social innovation, and acceptance of transition measures. Collective action in the energy transition is experimenting successfully with new business models in the energy sector^22–24^. Notably, financial data collected in the inventory allow for a more detailed analysis for some countries, although reporting obligations and practices differ. Those citizen initiatives that publish financial reports, tend to do so in more detail than incumbent enterprises, hence contributing to higher transparency. Our inventory data allow us to analyze financial performance and investment decisions by these initiatives compared to the overall performance of established enterprises. For example, Wierling et al*.*^24^ identify 9 successful business models for German initiatives active in the PV sector. Nevertheless, a comprehensive analysis across countries, new market actors, and fields of activities remains for future research. Thus, the dataset provides a unique and novel opportunity to study such questions.

Citizen initiatives are expanding their activities, as also evidenced in our inventory. Emerging fields for citizen action include community storage, e-mobility, virtual power plants^25^, community-hosted and community-developed open software platforms and one-stop shops (e.g., on demand-response or energy efficiency, see also^26,27^). While these activities are still relatively niche, we can report, for example, 182 initiatives active in energy storage in Europe.

Continued decentralization of energy systems and more stringent decarbonization policies will increase the importance of these actors in the future. Citizen-led energy action has already played, and will continue to play, an important role. This deserves systematic statistical accounting in addition to single case studies which currently dominate the literature (c.f. case studies for Spain:^28^, Austria:^29^, Ireland:^30^, UK:^31^, Sweden:^32^, Italy:^33^, Netherlands:^34^, France:^35^, Germany:^36^, and a rare study covering 16 countries^37^) and are alone insufficient to grasp the scope, extent, and future potential of citizen-led energy action^13,38^. As their total contributions to the low carbon transition have not been consistently and comprehensively estimated before, this study provides the first systematic aggregates at national and European scales, with such detail as is currently available. However, it should be recognized that substantially more work, automated data-mining, and standardized approaches will be needed to develop solid, intercomparable statistics.

## Methods

Citizen-led energy initiatives are organizations, formal or informal groups, or projects housed within some larger entity that fulfill (to greater or lesser degrees) each of the following criteria: (1) citizen leadership, (2) non-economic benefits, and (3) active in energy services provision. Citizen leadership means that the initiatives are led by physical persons or by organizations who are themselves citizen-led and are independent in operations and governance from for-profit private businesses or governments. Implicit in this criteria is adherence to the One-Member-One-Vote principle, although we find variations. The second criteria requires that the initiatives either do not pursue profit for their members, or, if profit is pursued, it is a means to another end, i.e. the stated goal is to redistribute social, ecological, and/or economic benefits to their community or wider society. The third criteria defines the scope of contributions to the energy transition that we estimate. Of interest are initiatives that engage in the production and distribution of renewable energy, invest in energy efficiency projects, and campaign or consult on all such activities, including education and awareness raising to foster behavior change towards a sustainable, low carbon energy transition. Organizations that meet all three criteria are the focus of the data presented here.

Notably, our dataset also includes some initiatives that meet the first two criteria, but are not primarily active in the energy sector, such as in the case of large-scale photovoltaic rooftops on agricultural cooperative buildings. Other initiatives that only partially meet one or more criteria have been included, in particular in countries where citizen-led energy ecosystems are emerging. The inclusion of these initiatives is intended to provide users of this dataset with a complete and inclusive perspective at a moment when each country is formalizing directives from the European Union that aim to increase the participation of citizens by providing them legal grounds to get involved^11,39^. While important for the coverage of this study, and significant on a country level, the inclusion of these border cases does not significantly alter the aggregate picture of the contribution of citizens in Europe to the energy transition.

The definition of "energy community" for most countries adheres closely to the existing cooperative legal structure, while countries such as Poland and Greece have taken markedly different approaches. In France, various types of organizations can be recognized as energy communities as it is not necessarily a distinct legal form. This results in a patchwork of definitions with some overlap across borders and forms. Additionally, while some basic administrative information (identification numbers, economic activity codes, addresses) can generally be found in a centralized national business register, the depth, breadth, and degree of accessibility of this data also differs from country to country. Only some countries maintain detailed, open, and up-to-date records of organizations' finances and activities based on legally required annual reporting. For others, we have had to rely on voluntarily shared data collected and centralized by umbrella organizations or on information taken from the websites and online publications of the initiatives themselves.

The large degree of variation in the quality and sources of data gathered resulted in an extensive, four-year long collection of data from thousands of sources through manual information gathering and compilation. Before the data collection step, meta-studies of the energy systems and policy contexts for each country were undertaken to identify pertinent legal forms, literature, and data sources. To increase comparability across countries, we have developed an ontology and set up internal accounting standards. To foster the reuse of data, the inventory adheres to the FAIR data principles^40^ which meant defining standards (e.g., for energy communities and their activities) where they do not exist. Data quality has been ensured by rigorous validation procedures, including the four-eyes principle, automated compliance checks, verification of data ranges, and, where possible, cross-checking of data with experts and against other publications and aggregated information sources. All data are published open-access with extensive documentation^41–43^. Details on aggregation methods and data collection for each country are described in the notes to the Supplementary Material (Supplementary Note 1, Supplementary Data 2 & 3).

## Supplementary Information

Supplementary Information 1.

Supplementary Information 2.

Supplementary Information 3.

## Data Availability

All data are available in the main text or the supplementary materials. The ENBP inventory "Energy by the People" is licensed under CC-BY 4.0 and available open access at dataverse.no, Link: https://doi.org/10.18710/2CPQHQ.
