# Supplementary material for: Statistical evidence for the contribution of citizen-led initiatives and projects to the energy transition in Europe
Source: Sci Rep. 2023 Mar 2;13:1342. doi: 10.1038/s41598-023-28504-4 (PMC9981751; doi:10.1038/s41598-023-28504-4)
Supplement: Supplementary file 1 — Supplementary Information 1. [file 41598_2023_28504_MOESM1_ESM.docx]

Supplementary Materials for

Statistical evidence for the contribution of citizen-led projects to the energy transition in Europe

Valeria Jana Schwanitz, August Wierling, Heather Arghandeh Paudler, Constantin von Beck, Simon Dufner, Ingrid Knutsdotter Koren, Tobias Kraudzun, Timothy Marcroft, Lukas Müller, Jan Pedro Zeiss.

Correspondence to: valerias@hvl.no

**This PDF file includes:**

Supplementary Note 1

**Other Supplementary Materials for this manuscript include the following (separate files):**

Supplementary Data 2_ENBP_Aggregates. Provides country- and EU-level aggregates (excel-file).

Supplementary Data 3_ENBP_Aggregates. Provides country- and EU-level aggregates (csv-file)

[**EU-level aggregation method**](#_heading=h.jvdqwbigaa9c) **2**

[**Country-level aggregation method**](#_heading=h.3avhrb7riz6k) **3**

[Austria (AUT)](#_heading=h.1zumdhyq3pg8) 4

[Belgium (BEL)](#_heading=h.lcgsimrmliwq) 6

[Bulgaria (BRG)](#_heading=h.a5ddiqfm2qqp) 7

[Croatia (HRV)](#_heading=h.fhny5dvycw6i) 8

[Cyprus (CYP)](#_heading=h.bmz9vd1uzi0w) 10

[Czech Republic (CZE)](#_heading=h.43hi2k56ximy) 11

[Denmark (DNK)](#_heading=h.ums4u336bzex) 12

[Estonia (EST)](#_heading=h.uhjlmtxq9ax0) 13

[Finland (FIN)](#_heading=h.crafu6eohl7t) 15

[France (FRA)](#_heading=h.wibck8we2nbg) 16

[Germany (DEU)](#_heading=h.cjkhbpkexcwq) 19

[Great Britain (GBR)](#_heading=h.9aef4s6h40gl) 21

[Greece (GRC)](#_heading=h.q2tau7hjdg2o) 22

[Hungary (HUN)](#_heading=h.w0fszqdgjoe4) 24

[Ireland (IRL)](#_heading=h.blwvoqw169me) 25

[Italy (ITA)](#_heading=h.ubpss13y3pn5) 26

[Latvia (LVA)](#_heading=h.1x04osdknwxr) 28

[Lithuania (LTU)](#_heading=h.jz8isiea2dad) 29

[Luxembourg (LUX)](#_heading=h.xnbgn59iq9e9) 31

[Malta (MLT)](#_heading=h.eichwc44sti2) 32

[Netherlands (NLD)](#_heading=h.635dx1jrbpyy) 33

[Norway (NOR)](#_heading=h.neuthyar58e5) 35

[Poland (POL)](#_heading=h.c55dgdq9jl37) 36

[Portugal (PRT)](#_heading=h.r0wlwfq4bdnu) 38

[Romania (ROU)](#_heading=h.g87lwz97z6di) 39

[Slovakia (SVK)](#_heading=h.pd9ymyu1fwkb) 40

[Slovenia (SVN)](#_heading=h.upbwvvr6ek5j) 41

[Spain (ESP)](#_heading=h.z7zco3mftdm) 43

[Sweden (SWE)](#_heading=h.yhdzhgd916c5) 44

[Switzerland (CHE)](#_heading=h.4ivhed7re0n3) 46

[**References**](#_heading=h.u8ahm2fq67f) **48**

# EU-level aggregation method

Estimates at the EU-level for the number of initiatives, people involved, projects, renewables capacities installed, and total funds invested are the sum of country-level estimates for the respective indicators. The results are reported in the Table S1 below. Details on the method for country-level estimates are described in the next section.

**Table S1.** Aggregate estimates for Europe.

| **Indicator** | **Quantitative estimate** | **Notes** |
| --- | --- | --- |
| Number of initiatives | 10,538 | Relevant initiatives fulfill three criteria to greater or lesser degree, i.e. the initiative is led by citizens, non-economic benefits are pursued in addition to economic benefits, and the initiative is active in the energy transition. The estimate counts the number of initiatives recorded in the inventory as well as the number of possible additional eco-villages and transition towns [2, 3]. The estimate is based on numbers from 30 countries. |
| Number of people involved | 2,010,602 | The estimate is based on available membership data from 29 countries. For initiatives not reporting on this data, we add estimates by assuming minimum and/or means while excluding outliers. Data from Cyprus is lacking. |
| Renewable capacities installed | 7.198-9.897 GW | Based on data from 29 countries. Low estimates assume 0% ownership, high estimates assume 100% ownership for those initiatives not reporting on ownership shares and it includes planned and capacities under construction for Croatia, France, Greece, Ireland, Netherlands, Poland and Spain. These capacities sum up to 876.5 - 991.5 MW. |
| Number of projects | 22,833 | The inventory has further details on 16069 production units and 625 equipment projects. Additional projects, such as investments into energy efficiency measures, are occasionally recorded. We also provide information on actual and planned fields of activities as announced on websites and/or statutes of initiatives (e.g., awareness raising events). The estimate is based on information from 30 countries. |
| Total funds invested | 6.2-11.3 Billion EUR | Low (high) estimates assume 0% (100%) ownership if data is not reported. In addition to reported investments, we estimate investments in production units if capacity values are known. We account for the change in prices if information on years is available. See country entries for methods and sources. The estimate is based on information from 25 countries (not available: Bulgaria, Czech Republic, Cyprus, Finland, and Norway). The high estimate includes investment estimates for planned and capacities under construction for Croatia, France, Netherlands, Greece, Ireland, Poland and Spain. |

# Country-level aggregation method

The results for the countries are presented in a similar fashion. In addition to national-level aggregates detailed in a table, we briefly review the current developments of citizen-led energy initiatives in the country. This includes a short statement of the progress of the implementation of the Renewable Energy Community (REC) and the Citizen Energy Community (CEC) definitions introduced by the Renewable Energy Directive (RED II) and the Internal Electricity Market Directive (IEMD). The update draws from country-level reports, scientific articles, and the REScoop Policy tracker [1]. A brief overview on fields of engagement listing areas of activities, starting with the most often reported, follows: This may include production of renewable-based electricity (various technologies), production of heat/biofuels, operation and management of distributions grids (electricity, heat, internet), mobility/transport (operation of charging stations, EV-car rental, etc.), energy efficiency measures, retrofitting of buildings, planning of renewable installations or energy efficiency equipment, consulting and financing of/on energy services, raising awareness, and information on energy transition and energy planning.

In addition, we report on how much (%) renewable energy makes up each country's final energy consumption, based on the latest country-specific updates from the IEA [2]. We include biofuels and waste, hydropower, and wind, solar, etc. as renewable energy sources. Also, we include the number of eco-villages, eco-cities, eco-communities, and/or transition towns that exist in specific countries from the Global Ecovillage Network (GEN) [3] and Transition Network [4] for aggregates, but do not include them in the database unless they have documented projects or other additional information. See Table S2 below for indicators and notes on derivation of quantitative estimates.

**Table S2.** Indicators and notes on derivation of quantitative estimates.

| **Indicator** | **Notes on derivation of quantitative estimate** |
| --- | --- |
| Number of initiatives | - Reporting number of initiatives as accounted for in the inventory. - Potentially add initiatives if numbers are known but further information is lacking, e.g., for eco-villages and as reported in peer-review literature or official reports. Inactive or dissolved initiatives are counted.   Judgment: 89% of the initiatives collected in our inventory report on the year of foundation, while dissolution years are seldom available, creating bias. This means that we underestimate the number of initiatives that may have existed, and been dissolved, at some point in the past. However, we are confident in our coverage of data available today. Note finally that many initiatives also only exist for a few years (reasons: only interested in running one project, merging with others or foundation of energy enterprise, or bankruptcy). |
| Number of people involved | - Sum of reported membership numbers - most recent years if membership is increasing. If numbers go up and down, the average of recent years is taken. - Additionally, membership numbers may be estimated for those initiatives not reporting, e.g., based on the average number or minimum number of members (case-by-case decision, exclusion of outliers), or on relevant ratios (e.g., members/equity, members/installed capacity, etc.). For housing associations, the number of apartments is used as a proxy. - Not counting the members of initiatives where the main purpose is not energy, e.g., forestry, agriculture or for eco-villages and transition towns, as information about the active participation of citizens is missing, and we do not have a solid ground for estimating.   Judgment: In all countries, reporting on the total number of members engaged in an initiative is purely voluntary. In some cases, these figures are centralized by umbrella organizations, but in most cases we had to rely on information gathered from initiative websites directly or from the meeting minutes of required yearly general assemblies. No effort at deduplication was attempted. While it is likely that some number of people are members of more than one organization within our database, no information permitting deduplication is available. Furthermore, it is felt that the conservative nature of the presented estimates regarding the number of people involved in an initiative when data was not present (i.e., the legal minimum in most cases), more than counterbalances this potential source of error. |
| Renewable capacities installed | - Reporting numbers for renewable-based capacities as recorded in the database: capacities for solar PV, solar thermal, wind, hydropower, geothermal, and heat. - Capacities planned or under construction are separately reported. - The numbers account for ownership by an initiative. For those initiatives where ownership shares are reported, we report the ownership-corrected value. For those initiatives not reporting on ownership, a low and a high estimate is provided, assuming 0% share and 100% share, respectively. In case of multiple ownership of an installation, the installation is only once counted. - Known capacities planned and/or under construction are added to the high estimate. - In some cases, renewable capacities are estimated when reliable averages for number and type of units per initiative can be made from the country data. |
| Number of projects | - Counting the number of projects mentioned by an initiative (planned, on-going, finalized). - For initiatives not reporting on any projects, at least one project is assumed. This is also the case for eco-villages and transition towns, etc. - Projects include both physical installations such as renewable energy production units as well as immaterial projects such as public education campaigns or consulting. |
| Total funds invested | - Total funds are the sum of funds reported, but investments before 1990 are excluded. - If investment data are not available, investments are estimated per renewable technology using turnkey prices for the respective year. The primary source of installation costs are country reports by the International Energy Agency, if those not available closest market data are used. For biomass based heat or electricity production, we refer to total investment costs as compiled by IRENA 2021 [103]. - Only including investments for which we have evidence that are energy-specific (e.g., excluding investments into agricultural production or forestry). |

## Austria (AUT)

The most common legal forms in Austria are *registrierte Genossenschaft* (m.b.H.) (registered cooperative) and *Verein* (association). The EU Directives on both RECs and CECs are implemented into national law. However, the national adaptation of the definitions pay little attention to the governance principles, citizen participation, and autonomy, indicating that the law still has some shortcomings. The historical evolution of the number of initiatives indicates that there has been a slow increase since the 1980s [5]. Additionally, 6 eco-villages, eco-cities, eco-communities, and/or transition towns exist in Austria.

Currently 33.8% of the country's final energy consumption is based on renewable energy [2]. The top-reported production technology utilized by initiatives is district heating (biomass) with 95% of initiatives in Austria being actively involved. Engagement in on- and off-shore wind energy and solar PV is only emerging. Other fields of engagement include hydro, cogeneration (biomass), electricity trade, providing broadband connection, e-mobility, solar thermal, and education and awareness raising. See Table S3 below for aggregate estimates of indicators for Austria.

**Table S3.** Aggregate estimates for Austria.

| **Indicator** | **Quantitative estimate** | **Notes** |
| --- | --- | --- |
| Number of initiatives | 389 | 383 registered energy cooperatives or energy cooperatives with limited liability (2 dissolved, 1 in liquidation). 6 eco-villages are added. |
| Number of people involved | 21750 | 48 initiatives report on members, the sum is 3651 (average: 76, median: 54). One initiative with ~400 members. 335 initiatives not reporting - added by assuming the median. Rounding. |
| Renewable capacities installed | 351.5 MW | Installed capacities sum up to 351.5 MW, thereof wind power 30 MW, solar PV and solar thermal 6 MW, hydro 14 MW, and biomass-based heat production and/or cogeneration 301 MW. All have 100% ownership. |
| Number of projects | 430 | 337 production units are reported. In addition, initiatives report on other activities. For eco-villages, we assume at least 1 project per initiative. |
| Total funds invested | 327.7 Million EUR | Investments in 27 production units - most of which were installed between 2000-2010 - sum up to 57.7 million EUR. Information on ownership is not available and thus assumed to be 100%. 309 production units do not report on financial investments (nor do initiatives report investments). Solar PV capacities built between 2015-2021 are accounted for with turnkey prices of 1200 EUR/kWp (sum of investments: 6.265 mill. Euro). Hydropower investments after 2005 are accounted for using the known costs for similar types with 2400 EUR/kW (sum of investments: 12.043 mill. EUR). Biomass-based heat/cogeneration for units installed after 2001 is estimated using the average from known investments (2000 EUR/kW, lower than IRENA [103] because of fuel costs not included) - sum of known capacities: 125.799 MW. The sum of investments: 251.74 mill. EUR. |

The following sources were used in determining the aggregates for Austria: [6—10]

AGRAR PLUS, "Heizwerk in Niederöstereich - Broschüre zu Heizwerken und erneuerbarer Energie in NÖ, Report 150" (Agrar Plus, nd; <https://agrarplus.at/folder-broschueren.html?file=files/agrarplus-inhalte/folder_broschueren/folder_150_heizwerk.pdf&cid=127>).

AGRAR PLUS, "Erneuerbare Energie in Niederösterreich, Report 200" (Agrar Plus, nd; <https://agrarplus.at/folder-broschueren.html?file=files/agrarplus-inhalte/folder_broschueren/folder200_heizwerk.pdf&cid=126>).

FirmenABC, Web service mirroring the Austrian Business Registry (2022). <https://www.firmenabc.at>.

Open Infra Map, Map of Austrian Power Plants (2022). <https://openinframap.org/stats/area/Austria/plants>.

M. Seiwald, The (Up) Scaling of Renewable Energy Technologies: Experiences from the Austrian Biomass District Heating Niche. *Moravian Geographical Reports*. **22/2**, 44-54 (2014). <https://doi.org/10.2478/mgr-2014-0011>.

## Belgium (BEL)

The most common legal forms in Belgium are *Société coopérative* (cooperative), *Société coopérative à responsabilité limitée* (limited liability cooperative), *Société coopérative à responsabilité limitée à finalité sociale* (social benefit limited liability cooperative), *Société coopérative européenne* (European cooperative), *Association sans but lucratif* (non-profit association), and *Association internationale sans but lucratif* (international non-profit association).

The implementation of the EU directives into national law is complete. However, Belgium's adaptation of the EU directives does not ensure citizen participation. The number of initiatives peaked in 2017, and there has been strong recent development due to good governmental support. Yet, there is a wide variety of regional specifications for citizen-led initiatives. Furthermore, 2 eco-villages and 1 transition town exist in Belgium.

Currently 10.2% of the country's final energy consumption is based on renewable energy [2]. Major fields of production technologies utilized by initiatives include on-shore wind (80%), solar PV (11%), and biomass based heat (8%). Other fields of engagement include hydro, e-mobility, cogeneration, biogas, biomass, electricity distribution and grid operation, and information and awareness services. See Table S4 below for aggregate estimates of indicators for Belgium.

**Table S4.** Aggregate estimates for Belgium.

| **Indicator** | **Quantitative estimate** | **Notes** |
| --- | --- | --- |
| Number of initiatives | 112 | 109 initiatives and 3 eco-villages/transition towns. The most common legal forms are 3 types of cooperatives. |
| Number of people involved | 162905 | 46 initiatives report on members (2014-2021), most recent summing up to 103055. Typical size: < 600, few >1000 members. One with ~60.000. Excluding the latter and assuming the average of 950 for those not reporting, adds 59850. |
| Renewable capacities installed | 155.6- 565.5 MW | 520 production units with installed renewable capacities are reported. Note that several units are co-owned by different initiatives. The low (high) estimate assumes 0% (100%) ownership if shares are not known.  Correcting for ownership, capacities add up to 128.9 MW (450.7 MW) onshore wind, 2.5 MW (62.5 MW) solar PV, 4.9 MW (5.7 MW) hydropower, and 19.3 MW (46.6 MW) biomass-based heat/cogeneration. |
| Number of projects | 850 | 520 production units in Belgium. Note that Belgium initiatives own 13 production units in Germany and 3 in France, whereas Switzerland owns production units in Belgium. In addition, 286 other projects. Assuming 1 project per eco-village. Rounding. |
| Total funds invested | 199.3-690.3 Million EUR | 124 data points for financial investments are available, summing up to 26.9-103.9 mill. EUR. The low (high) estimate assumes 0% (100%) ownership for initiatives not reporting. In addition, we estimate investments based on reported capacities if these are not yet included in above investments. For solar PV <250 kWp, we assume 100% ownership and account for price change in different years [2], yielding 21.6-116.0 mill. EUR. For wind, Duffy [45] cost-per-capacity figures for Germany were used, i.e. 1500 EUR/kW., leading to a range of 150.8-470.4 mill. EUR. No attempt is made to estimate costs for other technologies. Note that financial data is available for those initiatives where reporting is required. This does not include non-for profit organizations. |

The following sources were used in determining the aggregates for Belgium: [11—16, 45]

T. Bauwens, B. Gotchev, L. Holstenkamp, What Drives the Development of Community Energy in Europe? The Case of Wind Power Cooperatives. *Energy Research & Social Science.* **13**, 136-147 (2016). <https://doi.org/10.1016/j.erss.2015.12.016>.

D. Holemans, K. Van de Velde, "Citizens Energy: Making Energy Democracy Happen" (Green European Foundation & OIKOS Report, 2019; <https://gef.eu/wp-content/uploads/2019/01/GEF_Oikos_Citizens-Energy_Print.pdf>).

B. Wilken, International Energy Agency (IEA), "National Survey Report of PV Power Applications in Belgium, 2018" (IEA Photovoltaic Power Systems Programme, 2018; <https://iea-pvps.org/wp-content/uploads/2020/01/NSR_Belgium_2018.pdf>).

International Energy Agency (IEA), "National Survey Report of PV Power Applications in France, 2019" (IEA Photovoltaic Power Systems Programme, 2019; <https://iea-pvps.org/wp-content/uploads/2020/09/NSR_France_2019.pdf>).

G. Limpens, H. Jeanmart, F. Maréchal, Belgian Energy Transition: What Are the Options? *Energies.* **13/1**, 261 (2020). <https://doi.org/10.3390/en13010261>.

S. Oberthur, Ó. Söebech, H. Derde, K. Jackers, J. Vanhoenacker, T. Vermeir, W. Vandorpe, D. Haverbeke, "Legislative Options and Obstacles for Energy Communities in Belgium: Summary of Key Issues Identified & Recommendations" (ROLECS Project, Task 2.3.1, 2020; <https://cris.vub.be/ws/portalfiles/portal/55931943/Energy_communities_in_Belgium_legal_analysis_Oct_2020.pdf>).

A. Duffy, M. Hand, R. Wiser, Land-Based Wind Energy Cost Trends in Germany, Denmark, Ireland, Norway, Sweden and the United States. *Applied Energy*. **277**, 114777 (2020). <https://doi.org/10.1016/j.apenergy.2020.114777>.

## Bulgaria (BRG)

New forms of citizen-led energy initiatives are in their infancy in Bulgaria and the topics of individual and collective prosumption are only emerging [17—20]. This recent development is reflected in the lack of implementation of the EU Directives, as there is no existing legislation for energy communities as of today. Alleviation of energy poverty and energy security are important motivations for enabling community energy initiatives in the country.

We identify one energy community established in 2021 [21] that is registered as a limited liability company (Дружество с ограничена отговорност). Aiming to engage in the trade of electricity, it is the first private platform working with licensed electricity suppliers in Bulgaria. In addition, there are 13 eco-communities and eco-villages that exist in Bulgaria. Another interesting example is the attempt to explore options for forming a cross-border cooperation between Burgas in Bulgaria and Nis in Serbia to co-own PV systems. This project, mPower, is financed by the Horizon 2020 Program of the European Union. Legal forms for cooperatives and social benefit associations also exist in Bulgaria.

Currently 19.3% of the country's final energy consumption is based on renewable energy [2]. See Table S5 below for aggregate estimates of indicators for Bulgaria.

**Table S5.** Aggregate estimates for Bulgaria.

| **Indicator** | **Quantitative estimate** | **Notes** |
| --- | --- | --- |
| Number of initiatives | 14 | The first registered energy community in Bulgaria and 13 eco-communities. |
| Number of people involved | 93 | Minimum number of people required to register a limited liability company, assuming 7 members per eco-community. |
| Renewable capacities installed | n/a | Initiative engages in electricity trade, no production. |
| Number of projects | 14 | Assuming at least 1 per initiative. |
| Total funds invested | n/a | No data available. |

The following sources were used in determining the aggregates for Bulgaria: [17—22]

M. Vladimirov, A. Georgiev, S. Kolarova, "Development of Small-Scale Renewable Energy Sources in Bulgaria: Legislative and Administrative Challenges" (Center for the Study of Democracy, 2018; <https://csd.bg/publications/publication/development-of-small-scale-renewable-energy-sources-in-bulgaria-legislative-and-administrative-chal/>).

I. Todorović, "Bulgarian Municipalities Advised on Possibilities for Energy Communities" (Balkan Green Energy News, 2020; <https://balkangreenenergynews.com/bulgarian-municipalities-advised-on-possibilities-for-energy-communities/>).

I. Todorović, "Niš is Empowering Citizens within its Energy Transition" (Balkan Green Energy News, 2021; <https://balkangreenenergynews.com/nis-is-empowering-citizens-within-its-energy-transition/>).

Ministry of Energy and Ministry of the Environment and Water, "Integrated Energy and Climate Plan of the Republic of Bulgaria 2021-2030" (Republic of Bulgaria, 2019; <https://ec.europa.eu/energy/sites/default/files/documents/bg_final_necp_main_en.pdf>).

Izgrei, All Electricity Suppliers in One Place (2022). <https://www.izgrei.bg/>.

European Commission, "Commission Staff Working Document, Assessment of the Final National Energy and Climate Plan of Bulgaria, SWD (2020) 901 Final" (European Commission, 2020; <https://ec.europa.eu/energy/sites/default/files/documents/staff_working_document_assessment_necp_bulgaria_en.pdf>).

## Croatia (HRV)

The most common legal form is a cooperative (*Zadruge/Zadruga*). A small number of cooperatives are also active in the energy sector. Citizen-led renewable energy initiatives are a recent phenomenon in Croatia, but the sector shows signs of potential future growth. The implementation of the EU Directives is ongoing, with the government currently proposing one definition of CECs that reflects the requirement of cooperative governance principles and EU definitions. However, ensuring citizen participation is not yet covered in the national law.

Starting with the first initiative documented in our dataset that was established in 2002, available information on various planned projects and feasibility studies may indicate a rise in the number of initiatives in the near future. So far, initiatives in Croatia take an integrated approach, where the energy activity is but one pursuit among others. Eco-villages are such an "integrated approach" example, and 2 eco-villages exist in Croatia. Furthermore, the initiative 'Energetska zadruga otoka Krka' (Energy independent Island Krk) can serve as a prototype with a diversity of projects ranging from waste recycling, e-mobility, renewable energy production to consulting activities. This is different from other countries which often focus on production and distribution of renewable energy as the main activity.

Currently 31.6% of the country's final energy consumption is based on renewable energy [2]. The top-reported production technologies utilized by initiatives are onshore wind (98%) and solar PV (2%). Other fields of engagement include biomass/biogas for heating, e-mobility, financing, and consulting. Projects are exploring a variety of services as testbeds. See Table S6 below for aggregate estimates of indicators for Croatia.

**Table S6.** Aggregate estimates for Croatia.

| **Indicator** | **Quantitative estimate** | **Notes** |
| --- | --- | --- |
| Number of initiatives | 15 | 13 initiatives (10 are active), plus an additional 2 eco-villages. |
| Number of people involved | 1300 | Member information for 4 initiatives (1 dissolved) - 1234. Assuming 7 members for non-reporting initiatives. |
| Renewable capacities installed | 10.45 MW | 1 wind farm (10.250 MW, 2018), 4 PV initiatives with data (200 kWp). Additionally up to ~50 MW as reported in a feasibility study stage. |
| Number of projects | 16 | 5 realized projects (1 wind farm, 4 PV installations), for those not reporting we assume at least 1 project. |
| Total funds invested | 21.94-71.94 Million EUR | 1 wind farm (2018, 100% ownership): 21.74 million EUR. PV: estimated from capacities assuming 1000 EUR/kW.The high estimate also includes the cost for the planned investment. |

The following sources were used in determining the aggregates for Croatia: [23—25]

J. Eichermüller, M. Furlan, K. Habersbrunner, Z. Kordić, "Energy Cooperatives - Comparative Analysis in Eastern Partnership Countries and Western Balkans" (Women Engage for a Common Future, Zelena Energetska Zadruga (ZEZ), 2018; <http://www.wecf.org/wp-content/uploads/2018/06/EnergyCoops_LongOnline.pdf>).

E. Jerkic, "Energy Coops in Croatia" (Zelena Energetska Zadruga, 2016; <https://www.irena.org/-/media/Files/>[IRENA/Agency/Events/2016/Jan/18/ZEZ-Energy-coops-in-Croatia.pdf?la=en&hash=E72E78396642F36E972B9EE840CD09745A419746](https://www.irena.org/-/media/Files/IRENA/Agency/Events/2016/Jan/18/ZEZ-Energy-coops-in-Croatia.pdf?la=en&hash=E72E78396642F36E972B9EE840CD09745A419746)).

M. Kirac, M. Furlan, R. Pašičko, "Green Power to the People – Croatian Perspective on Community Energy" (Heinrich Böll Stiftung Belgrade, 2018;

<https://rs.boell.org/en/2020/04/10/green-power-people-croatian-perspective-community-energy>).

## Cyprus (CYP)

New forms of citizen-led energy initiatives currently do not exist in Cyprus (no database entries). The topics of individual and collective prosumption are only emerging [26, 27]. The government is making progress on the implementation of EU Directives that target the removal of financial and legal barriers for citizens to engage in the energy transition [28]. Cyprus currently does not have any legally recognized forms of cooperatives yet. However, according to the Cyprus Energy Agency (CEA) [26], it is expected that the first energy communities will soon be able to be established in Cyprus, supporting the process of building up knowledge with guidelines and manuals. This is reflected in the transposition process of the EU Directives, which has started but not yet completed. As the status of initiatives is none (early 2022), there are still several criteria that have yet to be addressed, such as citizen participation and inclusion of legal entities. Still, in Cyprus 1 eco-village exists as a pilot village and 1 transition town is planned, but further data is unavailable [3].

The energy system in Cyprus is still centralized with 100% of the electricity generation being owned by the Electricity Authority of Cyprus (EAC) [27]. This is seen as the main barrier toward citizen engagement. At present, 12% of the country's final energy consumption is based on renewable energy [2]. See Table S7 below for aggregate estimates of indicators for Cyprus.

**Table S7.** Aggregate estimates for Cyprus.

| **Indicator** | **Quantitative estimate** | **Notes** |
| --- | --- | --- |
| Number of initiatives | 2 | 2 eco-villages/transition towns. |
| Number of people involved | n/a | n/a |
| Renewable capacities installed | n/a | n/a |
| Number of projects | 2 | Assuming at least 1 project per initiative. |
| Total funds invested | n/a | n/a |

The following sources were used in determining the aggregates for Cyprus: [3, 26—28]

Global Ecovillage Network (GEN), Ecovillages Projects page (2022). <https://ecovillage.org/projects/>.

Cyprus Energy Authority (CEA), Energy Communities (2021). [https://www.cea.org.cy/en/energeiakes-koinotites](https://www.cea.org.cy/en/energeiakes-koinotites/).

Electricity Authority of Cyprus (EAC), Non Regulated Activities (2021). <https://www.eac.com.cy/EN/NonRegulatedActivities/about/Pages/default.aspx>.

European Commission, "Commission Staff Working Document, Assessment of the Final National Energy and Climate Plan of Cyprus. SWD (2020) 912 Final" (European Commission, 2020; <https://ec.europa.eu/energy/sites/default/files/documents/staff_working_document_assessment_necp_cyprus_en.pdf>).

## Czech Republic (CZE)

Few new forms of citizen-led energy initiatives currently exist in the Czech Republic, as the topics of individual and collective prosumption are just emerging. The implementation of EU Directives is ongoing and includes measures to reduce administrative burdens and enable a framework for citizen-led renewable energy initiatives.

The most common legal forms in Czech Republic are *Družstvo* (cooperative) and *Zemˇedˇelské družstvo* (collective farm). The vast majority of these initiatives are agricultural cooperatives, which recently also engage in biogas production and renewable-based electricity production. Many production units were commissioned between 2008-2011, and the earliest ones date from 1985. It is likely that other similar initiatives exist, but access to data is poor. In addition, 2 eco-villages exist in the Czech Republic.

Currently 15.8% of the country's final energy consumption is based on renewable energy [2]. Most initiatives engage in cogeneration with biogas. Other reported activities include solar thermal and electricity production based on solar PV and wind. See Table S8 below for aggregate estimates of indicators for the Czech Republic.

**Table S8.** Aggregate estimates for Czech Republic.

| **Indicator** | **Quantitative estimate** | **Notes** |
| --- | --- | --- |
| Number of initiatives | 38 | Few new forms of citizen-led energy initiatives, the majority are cooperatives that also engage in the energy transition. Out of the 36, 4 have been dissolved or have an unclear status. Additional 2 eco-villages. |
| Number of people involved | 266 | Minimum membership for coops and eco-villages: 7, rounding. |
| Renewable capacities installed | 30.9 MW | Data for 37 production units available, thereof 25.897 MW cogeneration, 3.8 MW from biomass-based heat production, 1 MW biogas, 52 kW solar thermal and 56 kWp solar PV. All ownership shares are assumed to be 100%. Capacity from planned wind park (39 MW) not included as citizen-involvement is limited to the possibility to invest in shares. Production data for 2 initiatives missing. |
| Number of projects | 42 | Typically 1 project/initiative, few have multiple projects. |
| Total funds invested | N/A | No attempt made to estimate. Market prices for installed capacities (cogeneration, etc.) are not available. Furthermore, only data about shares are available, but it is not possible to relate them to energy investments as the initiatives are mostly agricultural cooperatives. |

The following sources were used in determining the aggregates for the Czech Republic: [29—31]

European Commission, "Commission Staff Working Document, Assessment of the Final National Energy and Climate Plan of Czechia. SWD (2020) 902 Final" (European Commission, 2020; <https://ec.europa.eu/energy/sites/default/files/documents/staff_working_document_assessment_necp_czechia_en.pdf>).

D. Frieden, A. Tuerk, C. Neumann, S. d'Herbemont, J. Roberts, REScoop.eu, "Collective Self-Consumption and Energy Communities: Trends and Challenges in the Transposition of the EU Framework. Working Paper" (REScoop.eu., 2020; <https://www.rescoop.eu/uploads/rescoop/downloads/Collective-self-consumption-and-energy-communities.-Trends-and-challenges-in-the-transposition-of-the-EU-framework.pdf>).

V. Malý, M. Šafařík, R. Matoušek, "Consumer (Co-)Ownership in Renewables in the Czech Republic" in *Energy Transition Financing Consumer Co-Ownership in Renewables*, J. Lowitsch Ed. (Palgrave Macmillan: Cham, 2019), pp. 201-222.<https://doi.org/10.1007/978-3-319-93518-8>.

## Denmark (DNK)

The most common legal forms in Denmark are *interessentskab* (partnership) and *andelsselskab med begrænset ansvar* (limited liability organization). Detailed information is restricted due to data legislation and privacy laws. Implementation of EU Directives into national law is complete. However, there are still some elements of the EU definition that are yet to be addressed, such as defined purpose and ensuring citizen participation.

Denmark experienced two historic waves of the foundation of wind power cooperatives: 1985–1992 and 1998–2002. From 2002 there has been a strong decrease in the foundation of new wind power cooperatives due to changes in the feed-in tariff scheme and the beginning of commercial offshore wind investments. Coops that had been driving onshore wind were not able to continue with offshore wind. In addition, 27 eco-villages, eco-cities, eco-communities, and/or transition towns exist in Denmark.

Currently 37.5% of the country's final energy consumption is based on renewable energy [2]. Top-reported production technologies used by initiatives are district heating (90%) and onshore wind (10%). See Table S9 below for aggregate estimates of indicators for Denmark.

**Table S9.** Aggregate estimates for Denmark.

| **Indicator** | **Quantitative estimate** | **Notes** |
| --- | --- | --- |
| Number of initiatives | 665 | 278 initiatives engage in district heating and 360 in wind technology. 27 eco-villages are known. |
| Number of people involved | 306650 | District heating: membership data not available, but customer data for 138 initiatives with 286 672 customers. Wind: membership data are available for all initiatives. However, these initiatives have a specific legal form of a 'partnership' and we use the number of partners as the proxy for the number of members - we count 19 788 partners. We assume at least 7 members per eco-village (~190). |
| Renewable capacities installed | 2 613 MW | Reported capacities: 278 initiatives engage in district heating with 395 units (thermal capacity: 2342 MW - ca. 75% of DNK installed capacities). 355 initiatives engage in wind projects with 166 units (restricted access to ownership data according to data license): 272 MW. Note that about as much as 15-40% of all turbines installed in the country are/were owned by coops. Capacities installed before 2001 sum up to 42 MW, 60 MW were added from 2001-2010, and 170 MW afterwards. |
| Number of projects | 600 | Reporting the number of projects, not accounting for projects that only have minority shares. Assuming at least one project per eco-village, rounding. |
| Total funds invested | 411-2377 Million EUR | Investment data are not reported. Estimates are based on reported capacities for wind, but we refrain from providing detailed estimates for district heating as the age and technical specifications differ widely. However, investments are substantial, at least exceeding 2 billion EUR as the sum exceeds 2 GW capacity. We add this number to the upper estimate only. Wind capacities have been added between 1982-2020. Wind energy investment costs assumed to be 1500 EUR/MW (2002-2010) and thereafter 1250 EUR/MW (based on Duffy [45]). Most capacities have been added around the year 2000. We assume 2550 EUR/MW for installations before 2001 (based on IEA [112]). |

The following sources were used in determining the aggregates for Denmark: [5, 32, 33, 45]

A. Wierling, V.J. Schwanitz, J.P. Zeiß, C. Bout, C. Candelise, W. Gilcrease, J.S. Gregg, Statistical Evidence on the Role of Energy Cooperatives for the Energy Transition in European Countries. *Sustainability*. **10**, 3339 (2018). <https://doi.org/10.3390/su10093339>.

L. Gorroño-Albizu, K. Sperling, S. Djørup, The Past, Present and Uncertain Future of Community Energy in Denmark: Critically Reviewing and Conceptualising Citizen Ownership. *Energy Research & Social Science*. **57**, 101231 (2019). <https://doi.org/10.1016/j.erss.2019.101231>.

F. Mey, M. Diesendorf, Who Owns an Energy Transition? Strategic Action Fields and Community Wind Energy in Denmark. *Energy Research & Social Science*. **35**, 108–117 (2018). <https://doi.org/10.1016/j.erss.2017.10.044>.

A. Duffy, M. Hand, R. Wiser, Land-Based Wind Energy Cost Trends in Germany, Denmark, Ireland, Norway, Sweden and the United States. *Applied Energy*. **277**, 114777 (2020). <https://doi.org/10.1016/j.apenergy.2020.114777>.

## Estonia (EST)

Few new forms of citizen-led energy initiatives currently exist in Estonia, due to the recent emergence of the topics of individual and collective prosumption. The implementation of the EU Directives is complete and necessary regulations are amended, but Estonia does not strongly emphasize citizen participation in its interpretation of the concept "energy community". This matches the high degree of centralization of the Estonian energy system. The establishment of support programs for energy communities are under development.

The most common relevant legal forms identified are apartment associations (*Korteriühistu*) and non-profit associations (*Mittetulundusühing*). Recently, these initiatives are also engaging in energy efficiency projects, retrofitting of buildings, renewable-based electricity production, or the sustainable energy transition in general (e.g., in the case of eco-villages, of which there are 3 established in Estonia). Furthermore, we report on the activities of 3 commercial initiatives (Tulundusühistu, Osaühing), including two energy cooperatives and one eco-community, which is why we consider them as relevant for the database.

Currently 31.3% of the country's final energy consumption is based on renewable energy [2]. Top-reported production technologies used by initiatives are onshore wind (98%) and solar PV (2%). Other fields of engagement include retro-fitting of apartment buildings, onshore wind, consulting, and information and awareness raising. The first recorded installations date from 2016 and were installed on apartment buildings. See Table S10 below for aggregate estimates of indicators for Estonia.

**Table S10.** Aggregate estimates for Estonia.

| **Indicator** | **Quantitative estimate** | **Notes** |
| --- | --- | --- |
| Number of initiatives | 132 | 96% apartment associations, 4 other energy initiatives and 3 eco-villages. There are many more eco-villages in Estonia, but data is scarce. |
| Number of people involved | 5350 | No data about membership, but the number of units and sqm of housing initiatives is available. The unit size ranges between 50-80 sqm, speaking for family-size apartment buildings. We assume at least one member per unit and for the other 5 non-housing initiatives, 7-10 members. Rounding. |
| Renewable capacities installed | 12.65 MW | Data available for 8 solar PV projects with 100% ownership (0.209 MW, size: 15-74 kWp). Assuming an average of 20 kWp for the remaining 122 not reporting, adds 2.44 MW. Wind: 10 MW. |
| Number of projects | 142 | Counting production units and for those not reporting, we assume one project per initiative. |
| Total funds invested | 9.5 Million EUR | Multiplying solar PV capacities with 1000 EUR/kW (2.44 mill. EUR). In addition, subsidized reconstruction and investment grants for retro-fitting of apartment buildings. Lack of data for wind park investment, but share capital of 7 mill. EUR known. The remaining is likely financed by grants and loans. Decent rounding to account for retro-fitting investments. |

The following sources were used in determining the aggregates for Estonia: [34—36]

Energiatalgud, Energiaühistud (2021). <https://energiatalgud.ee/Energia%C3%BChistud?category=736>.

European Commission, "Commission Staff Working Document, Assessment of the Final National Energy and Climate Plan of Estonia. SWD (2020) 905 Final" (European Commission, 2020; <https://ec.europa.eu/energy/sites/default/files/documents/staff_working_document_assessment_necp_estonia_en.pdf>).

E. Risthein, T. Vaimann, *Eesti energeetika 100 aastat*. Post factum (2018).

## Finland (FIN)

Due to the recent emergence of the topics of individual and collective prosumption, few new forms of citizen-led energy initiatives currently exist in Finland. The implementation of the EU Directives is underway to remove technical and legal barriers for citizens to engage and are ongoing. However, there is currently no progress update available in the REScoop policy tracker [1].

In Finland, the most common legal form to involve citizens is a cooperative (*Osuuskunta*). Also relevant is the so-called "Mankala Principle" which may even have a larger impact on the citizen energy sector in the future. This principle exempts limited liability companies from paying taxes if it produces energy (or any other type of goods) for its shareholders at production cost without generating any profit. The shareholders of such an energy production company are required to pay for the operation costs of the Mankala company based on the percentage share they own. In turn, the shareholders have a right to the same percentage share of the energy produced by the company [37, 38]. Some of Finland’s nuclear power production companies are run according to this concept [38]. As a result, Finnish electricity prices, relative to the country's purchasing power standard, rank among the lowest in comparison to other European countries [39].

An outlier among the initiatives is "Metsäliitto Osuuskunta", a forestry and wood processing cooperative, which has installed several biomass cogeneration plants using waste products from its wood processing for energy self-consumption. The cooperative is by far the largest initiative in our dataset, with over 100,000 members and a yearly turnover of about 2 billion Euros. The yearly production of about 29 TWh equals 15% of the total yearly renewable energy generation in Finland [40].

Currently 45.8% of the country's final energy consumption is based on renewable energy [2]. Top-reported production technologies used by initiatives are district heating (45%), onshore wind (45%), biomass based heat (9%), hydro (<1%), and solar (<1%). Other reported fields of engagement include consulting and electricity-trading. In addition, 11 eco-villages, eco-communities, eco-cities, and/or transition towns exist in Finland. See Table S11 below for aggregate estimates of indicators for Finland.

**Table S11.** Aggregate estimates for Finland.

| **Indicator** | **Quantitative estimate** | **Notes** |
| --- | --- | --- |
| Number of initiatives | 94 | Cooperatives most common: 70 active, 13 dissolved. 11 eco-villages/ eco-communities/eco-cities. |
| Number of people involved | 105700 | 6 initiatives report on members (1 >103000, 1 ~ 1000, 1 ~ 440, 2x80, 1x50 members). Members assumed for coops not reporting: 7. Rounding. |
| Renewable capacities installed | 86.619-171.779 MW | 14 initiatives report on production data (43 units, 40 with capacity information), thereof, district heating 78.53 MW, heat boiler 15.65 MW, hydropower 0.55 MW, solar PV 70.06 MW, wind 77 MW (73 MW without ownership information). One of the projects is managed by the initiative and not the owner (6460 kW). Altogether, for 8 initiatives data on ownership is missing (3 of them also do not report capacity data). The lower estimate assumes 0% ownership shares for those not reporting; the upper estimate assumes 100%. |
| Number of projects | 120 | 43 production units from 13 initiatives (note: 9 additional projects to the 43 are an oil-based backup for an otherwise biomass-based production unit). Assuming at least one project for those not reporting and for eco-villages. Rounding. |
| Total funds invested | n/a | Only information on turnover available. No attempt made to estimate the financial contribution as most units concern heat installations. |

The following sources were used in determining the aggregates for Finland: [37—44]

Chambers & Partners, Finland: An Introduction to Banking & Finance (2021). <https://chambers.com/content/item/3909>.

J. Korteniemi, "Mankala Principle: A Concept to Finance Large Clean Energy Investments in Finland" (Finland Ministry of Economic Affairs and Employment, 2018; <https://www.ifnec.org/ifnec/upload/docs/application/pdf/2018-11/s3_3_fin_korteniemi.pdf>).

Eurostat, Electricity Prices for Household Consumers - Bi-Annual Data (from 2007 onwards) (Eurostat, 2021; <https://appsso.eurostat.ec.europa.eu/nui/show.do?dataset=nrg_pc_204&lang=en>).

Metsä Group, Metsä Group homepage (2019). <https://www.metsagroup.com>.

K. Huhtala, "Cooperatives in Finland –What they are Like and How they Operate" (2016; <https://www.chrissmithonline.co.uk/files/kari_edinburgh_2_2016.pdf> (link broken)).

O. Lehtonen, L. Okkonen, Energy Cost Reduction Creates Additional Socioeconomic Benefits– The Case of Eno Energy Cooperative, Finland. *Energy Policy*. **129**, 352–359 (2019).<https://doi.org/10.1016/j.enpol.2019.02.018>.

S. Ruggiero, A. Isakovic, H. Busch, K. Auvinen, F. Faller, "Developing a Joint Perspective on Community Energy: Best Practices and Challenges in the Baltic Sea Region" (Co2mmunity project, Working paper No. 2.3, 2019; <http://co2mmunity.eu/wp-content/uploads/2019/03/Co2mmunity-working-paper-2.3.pdf>).

Statistics Finland, Standard Industrial Classification TOL 2008 (Stat.fi, 2008; <https://www.stat.fi/en/luokitukset/toimiala/>).

## France (FRA)

The most common legal forms in France are SAS *société par actions simplifiée* (simplified joint-stock company), SCIC SAS (simplified joint-stock multi-stakeholder cooperative), SCIC SARL (limited liability multi-stakeholder cooperative), SCOP SARL *coopérative ouvrière de production* (worker cooperative), *Association déclarée* (association), *Association déclarée, reconnue d’utilité publique* (registered public benefit association), *Association non déclarée* (undeclared association), SICA SARL *d’intérêt collectif agricole* (collective interest limited liability agricultural company), *Société coopérative agricole* (agricultural cooperative), *Indivision entre personnes physiques* (partnership between physical persons), Autre SA *coopérative à conseil d’administration* (other joint-stock cooperative), and *autre* SARL *coopérative* (other limited liability cooperative).

Implementation of the EU directives into national law is nearly complete and assessed as good practice. However, there are some key issues yet to be addressed in the national definition, such as ensuring citizen participation and appointing an authority to oversee that the criteria are practiced lawfully. A final application decree establishing the details of geographic boundaries for energy communities and the definition of effective control is set to be published in 2022.

The current wave of citizen-led initiatives began in the early 2000s and evolved alongside an increase in the number of legal forms, both cooperative and non-cooperative. The highest number of new project foundations occurred between 2010 and 2017. The development of solar PV, which represents the majority of citizen-led initiatives in France, has slowed with the continued reduction of feed-in tariffs in France, and they are currently looking for new adaptive business models, such as collective consumption experiments.

Currently 15.5% of the country's final energy consumption is based on renewable energy [2]. Typical fields of engagement include the production of electricity or heat (onshore wind (70%), solar PV (27%), biomass based heat (3%), and hydro (1%, including a few older dams owned by historic rural electric coops and mostly modern microhydro)), and supply of energy and gas in general. Other reported fields of engagement include renovation, energy education, mobility, financing, consulting, and R&D - both social and technological. Most initiatives that engage in production specialize in one technology, and many participate in education and awareness and consciousness raising as well. Associations only very rarely participate in production and mainly carry on non-production activities. An additional 28 eco-villages, eco-cities, eco-communities, and 2 transition towns exist in France. See Table S12 below for aggregate estimates of indicators for France.

**Table S12.** Aggregate estimates for France.

| **Indicator** | **Quantitative estimate** | **Notes** |
| --- | --- | --- |
| Number of initiatives | 379 | 349 (mostly SAS, SCIC, SCOP, associations; most local distribution companies organized as SICA; most that engage in production activities organized as SAS or SCIC), 28 eco-villages/eco-communities, and 2 transition towns. |
| Number of people involved | 130000 | About half of the initiatives (excluding associations) engaged in production report member figures. 192 data points are available, summing up to 126350 members. This roughly breaks down to 55,000 Enercoop, 40,000 la Nef Cooperative Bank, 18,000 currently producing initiatives, 8,000 other and developing initiatives, 7,000 local distribution companies, 2,000 mobility. The final estimate is based on an average generated via a synthetic distribution using a representative sample of 163 energy community type initiatives engaged in production that report member figures applied to 33 such initiatives with no membership number available. The figure excludes 105 associations, most with no listed member data. While La Nef cooperative bank is not primarily focused on the energy transition, it is a founding partner of the major French umbrella organizations in the citizen energy movement and is often involved in funding larger installations. |
| Renewable capacities installed | 139-319 MW | Production data is relatively complete when compared with estimates by an in-country umbrella organization. 906 production units are reported in the inventory. The estimate for capacities is based on reported data for installation size. Note that the ownership percentage by citizen actors is accounted for. If we have no ownership information, it is assumed to be 0% for the lower bound and 100% for the upper limit. The lower (upper) bound is without (with) planned installations. Solar PV: 25-77 MW, wind onshore: 105-230 MW, hydro: 2.4-4 MW, wood-based heat and cogeneration: 6.2-7.5 MW. Note that these present ca. 0.2% of French installed RE electric capacity of 55.3 GW (excluding planned capacities of ~85-165 MW). We refrain from converting biogas capacities as these are minor. Thereof, 28 MW are owned by Swiss initiatives (wind onshore: 27.06 MW); hydro (lacking ownership information, we add 0 MW to our low estimate and 1.185 MW to our high estimate). |
| Number of projects | 2010 | 904 installed units (829 PV units, 30 wind units, 13 hydro, 5 biogas, 26 heat boilers, 1 cogeneration), 190 planned installations, 840 non-production projects. Aggregates with unknown unit numbers are assumed to have 1 project. We assume at least 1 project per eco-village/transition town. |
| Total funds invested | 204-455 Million EUR | About ⅓ of the units report on investments (accounting for ownership): solar PV 22-30 mill. EUR, wind 118-130 mill. EUR, hydro 4 mill. EUR, wood heat 4 mill. EUR, biogas 6 mill. EUR, cogeneration 1 mill. EUR. In addition to reported values, we estimate missing investments based on capacities reported, resulting in additional 17 million EUR for solar PV, 27 mill. EUR for wind, and 5 mill. EUR for heat-based installations. Solar PV cost estimates are taken from IEA country reports if n/a, with yearly/kW turnkey investment costs increased by 20% as inventory-derived figures showed that initiatives seem to pay a premium. Wind energy investment costs assumed to be the same as DEU for these periods, using Duffy [45]. Heat cost estimates use inventory-derived averages. Finally, including higher estimates in the high value reported in the quantitative estimate column, we also report on planned installations, sums: solar PV 19-45 mill. EUR, wind 96-174 mill. EUR, heat 2 mill. EUR, biogas 10 mill. EUR. Investment estimates not attempted for hydro or biogas, as reported data are too scarce and reliable cost estimates are not found. As a check on these investment figures, we take the median highest yearly equity+debt value for 129 representative initiatives (excluding Local Distribution Companies, Enercoop, and Energie Partagée Investissement) where we have this data, roughly 1 mill. EUR, and multiply by the number of economically productive initiatives (224). We arrive at a total figure of 224 mill., suggesting that our investment estimate is realistic and conservative. Mobility-related investments are not included in these figures as data was not available on an individual project basis. The sum of 2019 total assets for the 8 mobility initiatives reporting financial data is roughly 8 mill.EUR. |

The following sources were used in determining the aggregates for France: [14, 45]

International Energy Agency (IEA), "National Survey Report of PV Power Applications in France, 2019" (IEA Photovoltaic Power Systems Programme, 2019; <https://iea-pvps.org/wp-content/uploads/2020/09/NSR_France_2019.pdf>).

A. Duffy, M. Hand, R. Wiser, Land-Based Wind Energy Cost Trends in Germany, Denmark, Ireland, Norway, Sweden and the United States. *Applied Energy*. **277**, 114777 (2020). <https://doi.org/10.1016/j.apenergy.2020.114777>.

## Germany (DEU)

The most common legal forms in Germany are *Eingetragene Genossenschaft* (registered cooperative company) and *Eingetragener Verein* (registered association). The latter often engage in the energy transition as Germany has not adopted the EU Directives into national law and has no legislation specifically targeting energy communities. However, German law has allowed for the establishment of cooperatives since the adoption of the Cooperative Act Law of 1889 (last amended 16 October 2006). Germany's peak in its number of citizen-led energy initiatives occurred in 2014. Only around 65% have continued investments thereafter, in large part due to changes in the country's feed-in tariff scheme. Since 2000, associations have started to engage in the energy transition, and this trend has increased again in recent years. Additionally, 40 eco-villages, eco-cities, eco-communities, and/or transition towns exist in Germany.

Currently 17.2% of the country's final energy consumption is based on renewable energy [2]. Top-reported production technologies used by initiatives include solar PV (81%), onshore wind (13%), biomass (4.8%), and hydro (0.6%). The majority of associations participate in solar PV installations. Numbers are based on cooperative projects that have a primary focus on the energy transition. See Table S13 below for aggregate estimates of indicators for Germany.

**Table S13.** Aggregate estimates for Germany.

| **Indicator** | **Quantitative estimate** | **Notes** |
| --- | --- | --- |
| Number of initiatives | 5015 | 4983, thereof 1424 cooperatives and 3559 associations. 32 eco-villages (additionally, not listed in the ENBP inventory). |
| Number of people involved | 391500 | Data points for membership information by year and cooperatives primarily active in the energy sector (e.g., not counting agricultural cooperatives) account to 5698. Taking the most recent year reported, 686 unique entries are available (48% coverage). The number of people involved sums up to 235622. Thereof, 21 initiatives stand out as they have more than 1000 members (together 127 480). We are confident to capture all of them. To estimate people involved for cooperatives not reporting, we take the average for the sub-sample that excludes the large initiatives. With 163 members on average, we therefore estimate an additional 120013 members. As a general note, membership data of associations are not systematically available. The German law requires a minimum number of 7 for registered associations. According to Duffy [45], the average number of members in associations is 80. However, most of the initiatives engage in the energy sector as a side activity (e.g., a sports association installing a solar panel on the sportshall), which is why we only assume active involvement in energy-related activities of only 10 members per initiative, adding 35590 to the aggregate. We assume an additional 7 members per eco-village. Rounding. |
| Renewable capacities installed | 2157-3279 MW | 11135 production units are reported in the inventory, for 10265 the main owners are known as reported in the registry "Markstammdatenregister" [55]. Capacities per technology are the following: Solar: 10152 production units with an average ownership of 99% and an average capacity of 120 kWp. Correcting for ownership shares, the lower (upper) value for solar is 888 MW (1030 MW), assuming 0% (100%) shares for those not reporting. Most PV capacities were added 2010-2012, but continued afterwards at an intermediate level. Wind: 656 production units of wind parks and single installed turbines by German initiatives in Germany (ranging from 2 kW to 72 MW). Ownership not known for 96 production units. The average capacity amounts to about 5.5 MW per plant (about 2-3 typical turbines). On average, initiatives hold 88% of a plant. Correcting total capacities for ownership shares, the lower (upper) amount for wind is 1105 MW (2085 MW), assuming 0% (100%) shares for those not reporting. Hydropower: 72 production units, ownership not known for 3 units. The average ownership is 99%, average capacity of a unit 146 kW. Correcting total capacities for ownership shares, the lower (upper) amount for hydropower is 9 MW (10 MW), assuming 0% (100%) shares for those not reporting. Biomass-based heat, cogeneration, and biogas: 285 production units, ownership known for ~100% of them. Capacities amount to 155 MW (low and high). Geothermal and solar thermal units concern minor capacity additions which we do not report here. As a general note, about 15 units are installed abroad by German initiatives, which amount to about 5 MW of the capacities.  68.5 MW installed in Germany are owned by Swiss initiatives - wind: 68-69.3 MW solar PV: 0-0.5 MW. |
| Number of projects | 11500 | 11135 units for the production of renewable capacities and 412 equipment. Whereas associations typically have just one project, energy cooperatives engage in several (on average 5 installations projects). Initiatives are also active in other fields. Among them, 51 low-carbon mobility counts, 210 on distribution (including grid operation, trade, self-consumption, storage) and 120 on consulting and financing. Rounding. |
| Total funds invested | 3152-4614 Million EUR | In Germany, financial investments are in general less well covered compared to financial contributions of members through membership fees and shares. Collected investment data for production units are: PV investments sum up to 1502 mill. EUR (1700 mill. EUR), low (high) estimate respectively. The value accounts for the yearly change in PV price developments but as most installations are of size 10-100 kW, we assume the corresponding prices. These were at about 5000 EUR/kWp in 2006, falling to 1270 EUR/kWp by 2014, and showing another drop to 1000 EUR/kWp from 2020. The dynamics underline that the large additions around 2010 make up the major share in investments due to the higher PV module costs. Note that as the recording of financial investments for PV units is limited (only 10% report), we estimate all investments based on capacities reported. Wind investments: 474 mill. EUR are reported (109 data points compared to 629 production units). For these investments, capacities sum up to 259-395 MW (low/high ownership share assumptions), suggesting about 1.2-1.8 mill. EUR/MW (which is in line with Duffy [45]). In addition, we estimate investments from remaining capacities of 779-1622 MW (low/high), yielding 1168-2432 mill. EUR. We use Duffy [45] for wind investment figures. Therefore, total wind investments amount to 1643-2907 mill. EUR. Investment data for hydropower and bio-based renewable production are not available. Wind energy investment costs are based on Duffy [45]. Hydropower: 1.1 mill. EUR (3 data points compared to 72 production units). Biomass based heat, cogeneration, and biogas: 3.503 mill. EUR (9 of 285 units) Geothermal: 0.22 mill. EUR (1 datapoint). Hydrogen investments: 2.3 mill. EUR (1 data point), light contracting 25 000 EUR (2 data points). We abstain from estimating additional capacity investments as they are relatively marginal. |

The following sources were used in determining the aggregates for Germany: [45—55]

A. Duffy, M. Hand, R. Wiser, Land-Based Wind Energy Cost Trends in Germany, Denmark, Ireland, Norway, Sweden and the United States. *Applied Energy*. **277**, 114777 (2020). <https://doi.org/10.1016/j.apenergy.2020.114777>.

D. Bauknecht, S. Funcke, M. Vogel, Is Small Beautiful? A Framework for Assessing Decentralised Electricity Systems. *Renewable and Sustainable Energy Reviews*. **118**, 109543 (2020). <https://doi.org/10.1016/j.rser.2019.109543>.

Bundesverband der Vereine und des Ehrenamtes e.V. (Federal Association of Clubs and Volunteers), Vereine in Deutschland page (2022). <https://bundesverband.bvve.de/vereine-in-deutschland/>.

L.H. Broska, It’s All about Community: On the Interplay of Social Capital, Social Needs, and Environmental Concern in Sustainable Community Action. *Energy Research & Social Science*. **79**, 102165 (2021). <https://doi.org/10.1016/j.erss.2021.102165>.

S. Centgraf, Supporting Civic Engagement in German Energy Cooperatives – Transdisciplinary Research Based on the Reflection of Individual Needs. *Energy Research & Social Science*. **44**, 112 (2018). <https://doi.org/10.1016/j.erss.2018.05.003>.

C. Herbes, B. Rilling, L. Holstenkamp, Ready for New Business Models? Human and Social Capital in the Management of Renewable Energy Cooperatives in Germany. *Energy Policy*. **156**, 112417 (2021). <https://doi.org/10.1016/j.enpol.2021.112417>.

B. Schmid, T. Meister, B. Klagge, I. Seidl, Energy Cooperatives and Municipalities in Local Energy Governance Arrangements in Switzerland and Germany. *The Journal of Environment and Development*. **29/1** (2019). <https://doi.org/10.1177%2F1070496519886013>.

J. Schwabe, The Evolution of Cooperative Electric Carsharing in Germany and the Role of Intermediaries. *Environmental Innovation and Societal Transitions*. **37** (2020). <https://doi.org/10.1016/j.eist.2020.08.007>.

D. Süsser, M. Döring, B.M.W. Ratter, Harvesting Energy: Place and Local Entrepreneurship in Community-Based Renewable Energy Transition. *Energy Policy.* **101**, 332 (2017). <https://doi.org/10.1016/j.enpol.2016.10.018>.

A. Wierling, J.P. Zeiss, V. Lupi, C. Candelise, A. Sciullo, V.J. Schwanitz, The Contribution of Energy Communities to the Upscaling of Photovoltaics in Germany and Italy. *Energies*. **14/8**, 2258 (2021). <https://doi.org/10.3390/en1408225>.

Core Energy Market Data Registry, (nd). [https://www.marktstammdatenregister.de](https://www.marktstammdatenregister.de/MaStR/Einheit/Einheiten/ErweiterteOeffentlicheEinheitenuebersicht).

## Great Britain (GBR)

The most common legal form in Great Britain is Community Benefit Society, registered under the Co-op and Community Benefit Societies Act. Some other forms also exist including company, community interest company, and LLP. Many citizen-led initiatives were founded between 2011 and 2015. The number of new foundations has declined from 2016 with approximately 10 dissolving between 2016 and 2020. The average lifespan for an initiative in the UK is 5-10 years. Recently there has been a new increase. These waves mirror changes to the country's feed-in tariff legislation. An additional 17 eco-villages, eco-cities, eco-communities, and 8 transition towns exist in Great Britain. The UK is not a part of the European Union and therefore not obligated to implement the EU directives.

Currently, 12.2% of the country's final energy consumption is based on renewable energy [2]. Top-reported production technologies used by initiatives include solar PV, wind, and hydro. Another reported field of engagement is energy consulting. See Table S14 below for aggregate estimates of indicators for Great Britain.

**Table S14.** Aggregate estimates for Great Britain.

| **Indicator** | **Quantitative estimate** | **Notes** |
| --- | --- | --- |
| Number of initiatives | 387 | 362 initiatives, of which 94 were dissolved during the past years. In addition, 25 eco-villages/transition towns. |
| Number of people involved | 67425 | 62% of the initiatives report on members, whose sum is 41353. The average number of members is 185, which we use to complete the estimate for those not reporting. We assume an additional 7 members per eco-villages/transition towns. Rounding. |
| Renewable capacities installed | 234.5 MW | Based on aggregate information from 141 reporting initiatives (39%). As the data is limited to aggregate information only, it is reported as equipment/comments in the database. The sum of reported installed capacities amounts to 234.505 MW. |
| Number of projects | 533 | Most initiatives report on at least one project. Altogether, 508 different projects are registered. We assume at least one project per eco-village. |
| Total funds invested | 260.5 Million EUR | 57% of initiatives report on their assets, which sum up to 218.84 mill. GBP (exchange rate: 0.84 GBP/EUR). No estimate for those not reporting. |

The following sources were used in determining the aggregates for Great Britain: [5, 56, 57]

A. Wierling, V.J. Schwanitz, J.P. Zeiß, C. Bout, C. Candelise, W. Gilcrease, J.S. Gregg, Statistical Evidence on the Role of Energy Cooperatives for the Energy Transition in European Countries. *Sustainability*. **10**, 3339 (2018). <https://doi.org/10.3390/su10093339>.

Cooperatives UK, (2022). <https://www.uk.coop/>.

Mutual Public Register, Financial Conduct Authority (2021). <https://mutuals.fca.org.uk/>.

## Greece (GRC)

A number of new forms of citizen-led energy initiatives are registered in Greece. The most common legal form is an energy community (Ενεργειακή κοινότητα), a specific sub-form of a cooperative and, hence, the OMOV-principle is adhered to as per legal form. However, an open dispute about existing energy communities in Greece is the question of to what extent these initiatives are truly citizen-led and not a vehicle for private companies to benefit from governmental support for energy communities [58, 59]. We note this discussion but have no ground to exclude individual initiatives. However, as we provide conservative estimates for citizen-led investments and the number of people involved, we do not count them toward these two aforementioned categories. Additionally, Greece has 14 eco-villages and eco-communities.

The implementation of the EU Directives into national law is ongoing, with no drafts publicly released yet. It is expected that the issues mentioned above are set to be revised and dealt with in the transposition process. Currently 18.5% of the country's final energy consumption is based on renewable energy [2]. The top-reported production technologies utilized by initiatives are onshore wind (56%), solar PV (34%), and hydropower (10%). Other fields of engagement include e-trade, unspecified heat or electricity production, biomass, and cogeneration. See Table S15 below for aggregate estimates of indicators for Greece.

**Table S15.** Aggregate estimates for Greece.

| **Indicator** | **Quantitative estimate** | **Notes** |
| --- | --- | --- |
| Number of initiatives | 192 | Energy communities: 167 active, 9 dissolved, 2 in liquidation, plus 14 eco-villages. Most founded between 2018-2020, plus 1 in 2010, and 1 in 2013. 7 coop company/assoc., 2 EC/limited liability coop, 65 EC, 9 limited liability cooperative society. |
| Number of people involved | 2120 | Member data for 4 initiatives (2018-2021): 707. For those not reporting, we assume 7 members minimum (including eco-villages). Rounding. |
| Renewable capacities installed | 0-85.821 MW | 8 initiatives report on 53 production (but 34 are registered for electricity production, likely they are in the planning phase), Solar PV: 29181 kWp, Wind: 47900 kW, hydro: 8740 kW. Almost all are in construction or under development (lower bound zero). We assume 100% ownership. |
| Number of projects | 240 | 53 production units are reported, for the remaining we assume at least one project per initiative: 184. |
| Total funds invested | 102.621 Million EUR | Hybrid hydro-wind: 37 mill. EUR. Solar PV (2 projects): 0.655 mill. EUR. Rest of investments estimated from capacity values assuming 750 EUR/kWp for Solar projects and 1200 EUR/kW for wind. |

The following sources were used in determining the aggregates for Greece: [58—61]

M. Tsagkari, How Greece Undermined the Idea of Renewable Energy Communities: An Overview of the Relevant Legislation. *Law Environment and Development Journal (LEAD)*. **17** (2020). <https://www.researchgate.net/publication/346096106_How_Greece_Undermined_the_Idea_of_Renewable_Energy_Communities_An_Overview_of_the_Relevant_Legislation>.

I. Douvitsa, The New Law on Energy Communities in Greece. *Cooperativismo e Economía Social*. **40** (2018). <https://doi.org/10.35869/ces.v0i40.1385>.

D. Frieden, A. Tuerk, "Deliverable 2.3: Regulatory Frameworks for Energy Communities in the Pilot Site Countries Croatia, Spain, Greece, Portugal and Slovenia, Shaping EU Framework Transposition and Project Implementation" (Compile Project, 2020; <https://www.researchgate.net/publication/346652927_Deliverable_23_Regulatory_frameworks_for_energy_communities_in_the_pilot_site_countries_Croatia_Spain_Greece_Portugal_and_Slovenia_Shaping_EU_framework_transposition_and_project_implementation>).

A. Vasilakis,​ C. Vrettos, K. Dimitris, A. Kontolati, G. Koukoufikis, M. Margosi, F. Palaiogiannis, "Mapping of Energy Communities in Greece. HP1AB-00256, Deliverable 1.1" (Green Peace, 2020; <https://www.greenpeace.org/static/planet4-greece-stateless/184045bd-mapping_of_energy_communities_v1.2.pdf>).

## Hungary (HUN)

Few new forms of citizen-led energy initiatives currently exist in Hungary, as the topics of individual and collective prosumption are only emerging. The implementation of the EU Directives is ongoing. The concept of community energy, as well as similar forms, have not yet formally been established in Hungary. The country aims at implementing the two concepts from the EU Directives on energy communities in one approach. Citizen-participation is not strongly emphasized. This matches the high degree of centralization of the Hungarian energy system.

A few small-scale projects have grown from existing community structures [62], but limited information does not allow us to clarify whether the criteria for citizen-led projects apply to them. Hungary has the tradition of housing and agricultural cooperatives, but information about their activities in the energy sector could not be identified. For these reasons, we can only report one initiative which installed solar PV capacities with the help of crowd-financing. 7 additional eco-villages and eco-communities exist in Hungary. Currently 13.6% of the country's final energy consumption is based on renewable energy [2]. See Table S16 below for aggregate estimates of indicators for Hungary.

**Table S16.** Aggregate estimates for Hungary.

| **Indicator** | **Quantitative estimate** | **Notes** |
| --- | --- | --- |
| Number of initiatives | 8 | 1 citizen-led initiative, plus 7 eco-villages. |
| Number of people involved | 65 | Reported members of the initiative on the website for 2017 (15). Initiative reports high fluctuations in the number of members. We assume 7 members minimum per eco-village. |
| Renewable capacities installed | 30 kW | Rooftop solar installations. |
| Number of projects | 8 | Assuming at least 1 project per initiative and eco-village. |
| Total funds invested | 22500 EUR | Estimate using capacity multiplied by 750 EUR/kW [78]. |

The following sources were used in determining the aggregates for Hungary: [62—64]

I. Capellán-Pérez, N. Johanisova, J. Young, C. Kunze, Is Community Energy Really Non-Existent in Post-Socialist Europe? Examining Recent Trends in 16 Countries. *Energy Research & Social Science*. **61**, 101348 (2020). <https://doi.org/10.1016/j.erss.2019.101348>.

European Commission. "Commission Staff Working Document, Assessment of the Final National Energy and Climate Plan of Hungary. SWD (2020) 916 Final" (European Commission, 2020; <https://ec.europa.eu/energy/sites/default/files/documents/staff_working_document_assessment_necp_hungary_en.pdf>).

International Energy Agency (IEA), "National Survey Report of PV Power Applications in Italy 2020" (IEA Photovoltaic Power Systems Programme, 2020; <https://iea-pvps.org/wp-content/uploads/2021/11/NSR_Italy_2020.pdf>).

## Ireland (IRL)

New forms of citizen-led energy initiatives exist in Ireland. Those with legal forms are "friendly societies" (54SK). While cooperatives in general are well established in Ireland [65], energy cooperatives are still in their early stages and more comprehensive data on their numbers and activities is lacking. Nevertheless, most of the energy cooperatives focus on awareness raising, consulting work, and energy efficiency improvements for their members and customers [66]. According to Community Power [67], the lack of electricity producing cooperatives stems from the legal and technical difficulties of connecting to the grid. Implementation of the EU Directives into national law is ongoing, targeting some of the mentioned gaps and allowing advantages to RECs that want to connect to the grid.

Another notable new form of citizen-led initiatives in Ireland are so-called Sustainable Energy Communities [68]. They are registered with and supervised by the Sustainable Energy Authority of Ireland (SEAI). This government body was created to oversee the energy transition in Ireland by working with individuals, communities, and businesses. Communities and community energy are frequent targets of SEAI schemes following directives from the government highlighting the role of communities in the energy transition.

A sustainable energy community (SEC) is an organized group headed by a representative committee of citizens from a community. The formal power of this committee (e.g., against SEAI), as well as its openness to new members, is not formalized. For this reason, we flag the initiatives only as partially citizen-led. A SEC is registered under the SEAI’s SEC Network. It can include any non-profit, charity, co-op, or other voluntary organization with at least four members that engages in energy-related initiatives. SECs are directed to create Energy Master Plans for their communities, and various activities toward sustainable energy are encouraged. However, most SECs are primarily interested in raising energy-saving awareness or auditing energy use.

Currently 12.3% of the country's final energy consumption is based on renewable energy [2]. The top-reported production technologies utilized by initiatives are wind (63%) and solar PV (37%). The fields of engagement for the few existing energy cooperatives are mainly onshore wind and solar PV. See Table S17 below for aggregate estimates of indicators for Ireland.

**Table S17.** Aggregate estimates for Ireland.

| **Indicator** | **Quantitative estimate** | **Notes** |
| --- | --- | --- |
| Number of initiatives | 565 | 547 SECs, 14 cooperatives, and 4 eco-villages. Note that we do not report on the status of SECs due to lack of information. |
| Number of people involved | 25000 | No data points, but over 25000 citizens engage in community energy networks (SEAI, electric energy online 2021). Note that SEC Aurivo Cooperative has been reported to have about 10000 members alone. |
| Renewable capacities installed | 13500 kW | Reported are 8500 kW (wind) and 5000 kWp (solar, planned). |
| Number of projects | 565 | Assuming at least one project per initiative. |
| Total funds invested | 1.8-20.3 Million EUR | Estimate based on 1.8 mill. EUR/MW for wind (2012). 1000 EUR/kWp for solar, which is only accounted for in the high estimate. |

The following sources were used in determining the aggregates for Ireland: [65—70]

The Irish Co-operative Organization Society (ICOS), Map of Co-operatives (2021). <http://icos.ie/find-your-co-op/>.

Sustainable Energy Authority of Ireland (SEAI), Energy Communities (2022). <https://www.seai.ie/community-energy/sustainable-energy-communities/>.

Community Power, Ireland (2021). <https://www.communitypower.eu/en/ireland.html>.

Sustainable Energy Authority of Ireland (SEAI), Search our Sustainable Energy Community Network (2022). <https://www.seai.ie/community-energy/sustainable-energy-communities/sec-map/index.xml>.

Electric Energy Online, More than 500 Communities are Now Part of SEAI's Sustainable Energy Community Network (2021). <https://electricenergyonline.com/article/energy/category/climate-change/82/889075/more-than-500-communities-are-now-part-of-seai-s-sustainable-energy-community-network.html>.

European Commission, "Commission Staff Working Document, Assessment of the Final National Energy and Climate Plan of Ireland. SWD (2020) 906 Final" (European Commission, 2020; <https://ec.europa.eu/energy/sites/default/files/documents/staff_working_document_assessment_necp_ireland_en.pdf>).

## Italy (ITA)

The most common legal forms of cooperatives in Italy are *Associazione* (association), *Società Cooperativa* (cooperative), *Società Semplice* (simplified company), *Società Consortile a Responsabilità Limitata* (limited liability consortium), *Società Cooperativa a Responsabilità Limitata* (limited liability cooperative), *Consorzio* (consortium), *Società A Responsabilità Limitata* (limited liability company), *Società Per Azioni* (joint-stock company), and *Cooperativa Sociale* (social cooperative). Implementation of the EU Directives into national law is complete. The updated legislation includes improvements concerning autonomy and effective control that was not previously addressed.

In addition to the continued activities of historical rural electrification cooperatives in the alpine region, we observe two major periods in the development of citizen-led initiatives in the energy transition in Italy. First, the period from 2008-2013 was marked by a large number of new local and regional project foundations benefiting from favorable feed-in tariff conditions. Second, after the elimination of this support scheme, we observe a concentration in the sector in which several larger initiatives were able to grow to national scale and purchase solar PV units on the secondary market while also developing new projects. Furthermore, the recent creation of self-consumption energy community experiments potentially represents a new wave of development.

Currently 17.3% of the country's final energy consumption is based on renewable energy [2]. Top-reported production technologies used by initiatives include biomass based heat (50%), solar PV (25%), and hydro (23%). Other fields of engagement include electricity trading, energy efficiency, car sharing, awareness raising, planning and consulting, and operating heat, electricity, and internet grids. See Table S18 below for aggregate estimates of indicators for Italy.

**Table S18.** Aggregate estimates for Italy.

| **Indicator** | **Quantitative estimate** | **Notes** |
| --- | --- | --- |
| Number of initiatives | 207 | The database includes information on 196 citizen-energy initiatives, thereof 51 renewable energy communities, 138 other initiatives, 18 eco-villages with detailed information (but in the country a total of 29 eco-villages are known). Among the initiatives in the database are also 108 historical ones which were founded 1894-1990. |
| Number of people involved | 79420 | Evidence was collected that during 2011-2021 at least 78694 people were involved as members in different initiatives that primarily engage in the energy sector. We take the most recent values (if the membership has been increasing) or an average if membership is not steady. Data are lacking for 119 initiatives (93 of those focus on energy activities). We assume a minimum of 7 members for those initiatives not reporting (7x93 plus eco-villages). Rounding. |
| Renewable capacities installed | 293-348 MW | 252 units with solar PV: 88.132 MW, 8 production units with solar thermal (but data only for 3): 1523 kW. 100 hydropower units with 79.534 MW (21 without data). 18 are reporting on ownership data which gives 14765 kW (low estimate, assuming 0% ownership for those not reporting) and 76226 kW (high estimate, assuming 100% ownership for those not reporting). 18 wind (3 not reporting, ownership known for 5, 3 from CHE): low estimate: 15.7 MW (assuming 0% ownership for not reporting initiatives), high estimate: 16.7 MW (assuming 100% ownership). 98 biomass-based heat or electricity production (missing data for 19): 172.380 MW - assuming 100% ownership for these initiatives as it is the common option. 2 units geothermal: 392 kW. If not stated otherwise, ownership is assumed as 100%. 12.5 MW are owned by Swiss initiatives. |
| Number of projects | 558 | 517 production projects (10 of them are under construction), ~30 additional counts of activity fields not related to activities realized with production units, plus 11 assuming 1 per eco-village with no data. |
| Total funds invested | 110.8 - 184.8 Million EUR | 27 of 252 solar PV units with investment data: 11.956 mill. EUR. 11 of 100 hydropower units: 44.63 mill. EUR. 3 of 15 wind units: 514 000 EUR. 6 of 99 biomass-based heat or electricity production: 28.628 mill. EUR. Energy saving: 50.000 EUR. No information on solar thermal. In addition to reported values, we estimate missing investments based on capacities reported, resulting in additional 22- 95 million EUR for solar PV and 3-4 mill. EUR for wind. The range reported assumes a low of 0% and a high of 100% citizen ownership when ownership division is unknown. Solar PV cost estimates are taken from IEA country reports if n/a. Wind energy investment costs assumed to be the same as DEU for these periods, using Duffy [45]. |

The following sources were used in determining the aggregates for Italy: [45, 54, 64, 71—74]

A. Duffy, M. Hand, R. Wiser, Land-Based Wind Energy Cost Trends in Germany, Denmark, Ireland, Norway, Sweden and the United States. *Applied Energy*. **277**, 114777 (2020). <https://doi.org/10.1016/j.apenergy.2020.114777>.

A. Wierling, J.P. Zeiss, V. Lupi, C. Candelise, A. Sciullo, V.J. Schwanitz, The Contribution of Energy Communities to the Upscaling of Photovoltaics in Germany and Italy. *Energies*. **14/8**, 2258 (2021). <https://doi.org/10.3390/en1408225>.

International Energy Agency (IEA), "National Survey Report of PV Power Applications in Italy 2020" (IEA Photovoltaic Power Systems Programme, 2020; <https://iea-pvps.org/wp-content/uploads/2021/11/NSR_Italy_2020.pdf>).

C. Candelise, G. Ruggieri, Status and Evolution of the Community Energy Sector in Italy. *Energies*. **13/8**, 1888 (2020). <https://doi.org/10.3390/en13081888>.

A. Grignani, M. Gozzellino, A. Sciullo, D. Padovan, Community Cooperative: A New Legal Form for Enhancing Social Capital for the Development of Renewable Energy Communities in Italy. *Energies*. **14/21**, 7029 (2021). <https://doi.org/10.3390/en14217029>.

M.L.D. Silvestre, M.G. Ippolito, E.R. Sanseverino, G. Sciumè, A. Vasile, Energy Self-Consumers and Renewable Energy Communities in Italy: New Actors of the Electric Power Systems. *Renewable & Sustainable Energy Reviews*. **151**, 111565 (2021). <https://doi.org/10.1016/j.rser.2021.111565>.

F. Spinicci, "La Cooperative di Utenza in Italia: Casi di Studio" (European Research Institute on Cooperative and Social Enterprises (Euricse), 2011; <https://www.euricse.eu/wp-content/uploads/2015/03/1323957261_n1894.pdf>).

## Latvia (LVA)

The most common legal form in Latvia is *Biedriba* (society). The probability is high that additional housing associations exist, but are not registered. Therefore, our data provide a conservative estimate. Comparison with gray literature shows that our data collection and assessment sufficiently captures the current situation in Latvia. The topic of community energy is only emerging and citizens face high financial, social, and organizational barriers. The implementation of the EU Directives into national law is in progress, enabling frameworks for renewable energy communities. However, citizen participation is not emphasized. Also, at least 2 eco-villages exist in Latvia with an additional one in the planning stage.

Currently 41.5% of the country's final energy consumption is based on renewable energy [2]. Reported fields of engagement include retro-fitting of buildings often addressing energy efficiency measures and heating based on renewable energy. See Table S19 below for aggregate estimates of indicators for Latvia.

**Table S19.** Aggregate estimates for Latvia.

| **Indicator** | **Quantitative estimate** | **Notes** |
| --- | --- | --- |
| Number of initiatives | 8 | Housing initiatives and 4 eco-villages/transition towns. The number is very likely much higher, as registration is not obligatory. |
| Number of people involved | 150 | One initiative with 42 apartments, another 33 apartments, and a third is a multi-family building (for which we assume 10 apartments). We take the average of these for assuming the number of apartments for the fourth initiative (28 apartments). Lower estimate for number of people involved, assuming 1 person/apartment. Minimum of additional 28 members are assumed for eco-villages. Rounding. |
| Renewable capacities installed | 130 kW | Lower range as reported. |
| Number of projects | 9 | Retro-fitting projects. Assuming at least 1 project per initiative, with 1 initiative currently having 2 active projects. |
| Total funds invested | 0.825 Million EUR | Reported investments only, as capacities installed are a conservative estimate. |

The following sources were used in determining the aggregates for Latvia: [43, 75—78]

S. Ruggiero, A. Isakovic, H. Busch, K. Auvinen, F. Faller, "Developing a Joint Perspective on Community Energy: Best Practices and Challenges in the Baltic Sea Region" (Co2mmunity project, Working paper No. 2.3, 2019; <http://co2mmunity.eu/wp-content/uploads/2019/03/Co2mmunity-working-paper-2.3.pdf>).

Co2mmunity, "Co2mmunity, Co-Producing and Co-Financing Community Energy Projects, Co2mmunity RENCOPs Summary Booklet, V1.0 (Co2mmunity, 2020; <https://co2mmunity.eu/wp-content/uploads/2020/10/2020_09_30_RENCOP_Booklet.pdf>).

Community Power, Latvia (nd). <https://www.communitypower.eu/en/latvia.html>.

European Commission, "Commission Staff Working Document, Assessment of the Final National Energy and Climate Plan of Latvia. SWD (2020) 913 Final" (European Commission, 2020; <https://ec.europa.eu/energy/sites/default/files/documents/staff_working_document_assessment_necp_latvia_en.pdf>).

E. Laes, K. Anfinson, M. Krug, V. Gatta, E. Meynaerts, E. De Luca, R. Cotroneo, M. Caliano, G. Klāvs, I. Kudrenickis, S. Aakre, H. Sælen, K. Standal, P. Nowakowski, R. Wnuk, I. Azevado, P. Maleki, Assessment Report of Potentials for RES Community Energy in the Target Regions, Deliverable 2.2 (COME RES Project, 2021; <https://come-res.eu/fileadmin/user_upload/Resources/Deliverables/Del_2.2_Assessment_Report_of_Potential.pdf>).

## Lithuania (LTU)

No specific legal forms for energy communities were identified in Lithuania; the general cooperative form exists though. The probability is high that additional housing associations exist that engage in energy, but are not registered. Thus, our data provide a conservative estimate. The topic of community energy is only emerging and citizens face high financial, social, and organizational barriers as confirmed by the group of experts. We concluded that the initial collection of a limited number of housing associations is justified. Additionally, 2 eco-villages/eco-communities exist in Lithuania.

Also, cooperative forms are still confronted with historical prejudices, originating from negative experiences during the socialist era [43]. The authors do not report any existing initiative. The implementation of EU Directives is complete with an emphasis on enabling citizen participation in REC. However, there is no CEC definition in the law yet, indicating there is still work to be done on the transposition.

Currently 33.5% of the country's final energy consumption is based on renewable energy [2]. Reported fields of engagement are connected to the renovation of heating and electricity supply in housing, including various technologies such as geothermal, solar PV, and biomass. See Table S20 below for aggregate estimates of indicators for Lithuania.

**Table S20.** Aggregate estimates for Lithuania.

| **Indicator** | **Quantitative estimate** | **Notes** |
| --- | --- | --- |
| Number of initiatives | 21 | 18 housing associations, 1 wind initiative, and 2 eco-villages/ eco-communities. |
| Number of people involved | 650 | Members of housing associations and 1 energy community. Apartments have between 30-63 units (average units: 53) reported for 9 of 11 housing associations). The size is 60-70 sqm per unit (family-size apartments). We assume one member per apartment as a lower estimate. We assume average values for the 2 associations without data. For the other initiatives we assume 10 members in each, and the wind association reports 40 members. Rounding. |
| Renewable capacities installed | 264 kW | Wind (250 kW, sold), geothermal (14 kW). Estimate based on BSR database [43]. |
| Number of projects | 21 | Assuming at least one project per initiative/eco-village. |
| Total funds invested | 4.860 Million EUR | Retrofitting investments are known for 8 of 11 housing projects, which are allocated to energy efficiency measures. Two reported investments for production units (one wind project). |

The following sources were used in determining the aggregates for Lithuania: [43, 79]

S. Ruggiero, A. Isakovic, H. Busch, K. Auvinen, F. Faller, "Developing a Joint Perspective on Community Energy: Best Practices and Challenges in the Baltic Sea Region" (Co2mmunity project, Working paper No. 2.3, 2019; <http://co2mmunity.eu/wp-content/uploads/2019/03/Co2mmunity-working-paper-2.3.pdf>).

European Commission, "Commission Staff Working Document, Assessment of the Final National Energy and Climate Plan of Lithuania. SWD (2020) 914 Final" (European Commission, 2020; <https://ec.europa.eu/energy/sites/default/files/documents/staff_working_document_assessment_necp_lithuania_en.pdf>).

## Luxembourg (LUX)

The most common legal forms in Luxembourg are *Société civile* (civil company), *Société coopérative* (cooperative society), *Association sans but lucratif* (nonprofit association), and *Société coopérative organisée sous forme de société anonyme* (cooperative company organized as a shareholder company). A few historical electric coops were founded in the beginning of the 20th century, while recently the sector has become more dynamic. According to the Bridge Report (2019), "a couple" of cooperatives engaging in the energy transition exist. We are able to identify 66 initiatives (44 citizen-led initiatives and 22 energy communities with limited citizen-leadership). In addition there is 1 eco-village and 1 transition town in Luxembourg.

The transfer of the EU Directives into national law is only proposed. Notably, some of the ICA/EU criteria have not been adopted, including autonomy and openness, and citizen participation is not ensured. Currently 16.5% of the country's final energy consumption is based on renewable energy [2]. The top-reported production technologies in Luxembourg utilized by initiatives include onshore wind (96%) and solar PV (4%). Other reported fields of engagement include biomass based heating, trade of electricity, energy services, consulting and awareness raising, and energy savings. See Table S21 below for aggregate estimates of indicators for Luxembourg.

**Table S21.** Aggregate estimates for Luxembourg.

| **Indicator** | **Quantitative estimate** | **Notes** |
| --- | --- | --- |
| Number of initiatives | 68 | 44 citizen-led initiatives, 22 energy communities with limited citizen-leadership, 1 eco-village, and 1 transition town. |
| Number of people involved | 1200 | Data for just 3 initiatives: 2 of them reporting 10-13 members (2020/2019), one reports 212-230 (2019/2020). We assume on average 15 members for the remaining 63 initiatives. Rounding. |
| Renewable capacities installed | 1.035-25.235 MW | Solar PV (1005 kWp + 30 kWp calculated from yearly production), heating (777000 kWh - not accounted for), wind (24200 kW). Ownership for wind is restricted data, assuming 0% (100% ) for the lower (upper) estimate. |
| Number of projects | 86 | 20 projects to install RE capacities are known, but only 10% of the initiatives report on projects in general, those reporting manage between 1-7 projects. We assume at least one project for those not reporting. |
| Total funds invested | 4.028 Million EUR | The capital reported by 27 initiatives is about 1.3 mill. EUR (61000 EUR potentially double counted, therefore subtracted). Investment data reported for 6 RE projects: 272000 EUR for wood-based heat generation/with solar, energy savings investments: 225000 EUR, 7020 EUR for wind project (share only), 165000 EUR for 160 kWp solar and 180000 EUR for 200 kWp solar - sum: 2.142 mill. EUR. In addition we estimate: for remaining solar PV projects realized in 2019/2020: 206.16 kWp x 1000 EUR/kWp and 668,7 kWp realized before 2015 x 2500 EUR/kWp. |

The following source was used in determining the aggregates for Luxembourg: [80]

Bridge Project, "Energy Communities in the EU - Task Force Energy Communities. Bridge Project, Report D3.12.d" (Bridge Project, 2019; <https://www.h2020-bridge.eu/wp-content/uploads/2020/01/D3.12.d_BRIDGE_Energy-Communities-in-the-EU-2.pdf>).

## Malta (MLT)

Cooperative Society is the most common legal form in Malta. The concept of community energy and other similar forms have not formally been established in Malta, although a tradition of cooperatives active in other fields exists.

We only report two initiatives, and one of them operates a PV project, for which we estimate the investment with the help of market prices for PV. The project was financed with the help of crowdfunding. No attempt is made to generate estimates/scaling up of other information. The overall legal and financial barriers to collective action in the country are high, and Malta prefers having a centralized organization of the energy system. For example, the requirement for a starting capital of 0.1 million EUR is seen as a barrier [81]. Therefore, emphasis is put on supporting individual self-consumption [82]. Interestingly, Malta has no intention of transferring the EU provisions on energy communities, with their NECP explicitly rejecting the development of energy communities. This is attributed to the lack of market (monopoly) for supply of energy. Currently 7.7% of the country's final energy consumption is based on renewable energy [2]. See Table S22 below for aggregate estimates of indicators for Malta.

**Table S22.** Aggregate estimates for Malta.

| **Indicator** | **Quantitative estimate** | **Notes** |
| --- | --- | --- |
| Number of initiatives | 2 | Identified cooperatives related to energy transition. |
| Number of people involved | 366 | Data on applicants invited to join for one of the projects. |
| Renewable capacities installed | 997 kWp | Solar PV, ownership assumed as 100%. |
| Number of projects | 2 | Assuming at least 1 project per initiative. |
| Total funds invested | 0.75 Million EUR | Using capacity data, multiplied by 750 EUR/kWp [64]. |

The following sources were used in determining the aggregates for Malta: [63, 64, 81—83]

European Commission. "Commission Staff Working Document, Assessment of the Final National Energy and Climate Plan of Hungary. SWD (2020) 916 Final" (European Commission, 2020; <https://ec.europa.eu/energy/sites/default/files/documents/staff_working_document_assessment_necp_hungary_en.pdf>).

International Energy Agency (IEA), "National Survey Report of PV Power Applications in Italy 2020" (IEA Photovoltaic Power Systems Programme, 2020; <https://iea-pvps.org/wp-content/uploads/2021/11/NSR_Italy_2020.pdf>).

C. Quintana, The Power of Cooperation, Cooperatives Europe Key Figures 2015" (Cooperatives Europe, 2016; <https://coopseurope.coop/sites/default/files/The%20power%20of%20Cooperation%20-%20Cooperatives%20Europe%20key%20statistics%202015.pdf>).

European Commission, "Commission Staff Working Document, Assessment of the Final National Energy and Climate Plan of Malta. SWD (2020) 917 Final" (European Commission, 2020; <https://ec.europa.eu/energy/sites/default/files/documents/staff_working_document_assessment_necp_malta_en.pdf>).

Malta Co-operative Federation, Co-operative Federation (2021). <https://maltacooperativefederation.coop/>.

## Netherlands (NLD)

The most common legal forms in the Netherlands are *Coöperatie* (Cooperative), *Vereniging* (Association, Society), and *Stichting* (Foundation, Trust). Initiatives since 2015 have focused on wind, with a peak in 2018. There has been an increase in the foundation of initiatives in recent years (see also Hufen *et al.* (2015) [84] and Boon *et al.* (2014) [85]). In addition, 8 eco-villages, eco-cities, eco-communities, and/or transition towns exist in the Netherlands. Currently 8.5% of the country's final energy consumption is based on renewable energy [2].

Fields of engagement include solar PV (92 %) and wind (8 %). Solar PV is also established on water. It is noteworthy that a large number of wind projects are currently in the planning stage. Assuming that all planned projects will be realized, this would almost double the total current capacity installed by citizen-led initiatives. As a result, the relative engagement in wind energy would drastically increase from the current 8%. Additional fields of engagement include energy coaching and consulting, energy efficiency, electricity trading, biogas trading, mobility, distribution networks & internet, banking and finances, and information and awareness raising. See Table S23 below for aggregate estimates of indicators for the Netherlands.

**Table S23.** Aggregate estimates for the Netherlands.

| **Essential information** | **Quantitative estimate** | **Notes** |
| --- | --- | --- |
| Number of initiatives | 999 | Energy cooperatives, foundations, and other associations contributing to the energy transition. 8 eco-villages and 1 transition town. We follow Hier Opgewekt [89] concerning the identification of relevant initiatives. |
| Number of people involved | 188400 | 170 initiatives are reporting membership data. These sum up to 44013 (thereof 14 414 from initiatives with members > 1000). Extrapolating from the mean of 177 based on a subsample for the majority of initiatives (<1000 members), yields a total of additional 144 320 members. We assume at least 7 members for eco-villages and transition towns. Rounding. |
| Renewable capacities installed | 613-1027 MW | 1049 production units are reported. 882 PV production units have detailed information about capacities and ownership shares for 773 units. Installed solar PV capacities sum up to 289-401 MW (lower value assumes 0% if information on ownership share is not available). 167 wind production units are reported. For 93 of them the installation has been finalized (3 are out of operation). 85 of them report all necessary information on capacities and ownership shares, summing up to a total capacity of 324 MW (lower value assumes 0% if information on ownership share is not available). In addition, about 591-626 MW of capacity are planned or in the process of installation, which we add to the higher value (also assuming 100% ownership for those initiatives not reporting shares). We know of about 3 additional heating projects, but information on capacities is lacking. |
| Number of projects | 1446 | More than half of the initiatives engage in the production of renewable energy capacities (~40% solar PV, 15% onshore wind). Electricity trading, energy consulting and energy efficiency are other important fields of activities (5-10% of initiatives report on them). 1052 reported installed production units, information. There are about 413 additional projects in the planning or implementation phase or concerning other activities (including 6 hydropower, 3 biogas and 39 heat projects). Regarding other activities, initiatives report projects on electricity trading (81), biogas trading (2), distribution networks & internet (8), energy consulting (52), energy efficiency (48), mobility (17) and banking and finances (2) and information and awareness raising (6). We assume one project per eco-village/transition town. |
| Total funds invested | 733-1282 Million EUR | Financial information is based on installed capacities estimated. Wind energy investments: about 30 installed projects with full information available where built before 2010. However, capital costs have been relatively stable across Europe between 2008-2016 (using Duffy [45], note that the values are not corrected for inflation). We use the data for Germany to provide the estimate. Assuming 1500 EUR/MW, we arrive at 486-939 mill. EUR (assuming 0% for ownership shares if they are not available). Solar PV investments: 95% have been added since 2015, which is why we refrain from differentiating investment costs for earlier years. We use investment costs for fixed solar PV installations as reported in Lugo-Laguna *et al*. (2021) [91]. Assuming 854 EUR per kW, we arrive at 247-343 mill. EUR (assuming 0% shares for the lower estimate). We refrain from estimating financial contributions to other projects. |

The following sources were used in determining the aggregates for the Netherlands: [45, 84—91]

A. Duffy, M. Hand, R. Wiser, Land-Based Wind Energy Cost Trends in Germany, Denmark, Ireland, Norway, Sweden and the United States. *Applied Energy*. **277**, 114777 (2020). <https://doi.org/10.1016/j.apenergy.2020.114777>.

J. Hufen, J. Koppenjan, Local Renewable Energy Cooperatives: Revolution in Disguise? *Energy, Sustainability and Society*. **5/1** (2015). <https://doi.org/10.1186/s13705-015-0046-8>.

F. Boon, C. Dieperink, Local Civil Society Based Renewable Energy Organizations in the Netherlands: Exploring the Factors that Stimulate their Emergence and Development. *Energy policy*. **69**, 297–307 (2014).<https://doi.org/10.1016/J.ENPOL.2014.01.046>.

Bedrijvenmonitor, Dutch Business Register (nd). <https://bedrijvenmonitor.info/>.

Energie Sammen, Leden en supporters (nd). <https://energiesamen.nu/pagina/1/jouw-belangenvereniging/35/leden-en-supporters>.

Esri Nederland, Windturbines (ArcGIS map containing information on wind turbines) (nd). <https://www.arcgis.com/home/item.html?id=4d2e288f6d26406aadba730414faf7a0>.

Hier Opgewekt, Lokale Energie Monitor 2020, Excel dataset (2021). <https://www.hieropgewekt.nl/lokale-energie-monitor>.

KVK Handelsregister Open Data Set (nd). <https://www.kvk.nl/producten-bestellen/koppeling-handelsregister/kvk-handelsregister-open-data-set/>.

D. Lugo-Laguna, A. Arcos-Vargas, F. Nuñez-Hernandez, A European Assessment of the Solar Energy Cost: Key Factors and Optimal Technology. *Sustainability*. **13**, 3238 (2021). <https://doi.org/10.3390/su13063238>.

## Norway (NOR)

The most common legal forms in Norway are *Samvirkeforetak* (cooperative) and *Europeisk samvirkeforetak* (European cooperative). Information is only available from one study of the Norwegian community energy sector conducted by the Norwegian Water Resources and Energy Directorate in 2018. This report lists general information only and concludes that detailed information is restricted due to data legislation and privacy. 5 initiatives were reported to be active with an additional 25 in implementation stages. Further details are not available. Only known information is reported without attempting to suggest estimates. The database provides the link to the report and counts projects and initiatives. In addition, Norway has 6 eco-villages and eco-communities that exist or are in planning stages.

Norway is not an EU Member State, but is a part of the European Economic Area (EEA). With the EU Directive on REC and CEC still under review by the EEA, the transposition timeline for Norway is likely long-term. Furthermore, there are some burdensome regulations in Norway, notably that joint metering is not allowed, which prevents energy community initiatives from starting up and being economically sustainable.

Currently 62.4% of the country's final energy consumption is based on renewable energy [2]. The top-reported production technologies utilized by initiatives are solar PV (14%) and wind (63%). Note that 22% of the recorded capacities have no technological specification. Other reported fields of engagement include energy efficiency, end-user flexibility, security of supply, and grid investments such as storage and trade. See Table S24 below for aggregate estimates of indicators for Norway.

**Table S24.** Aggregate estimates for Norway.

| **Indicator** | **Quantitative estimate** | **Notes** |
| --- | --- | --- |
| Number of initiatives | 36 | 5 active local energy communities and 25 local energy communities in development (status 2018). Driven by property developers and distribution network operators. Data is fully anonymized. An additional 6 eco-villages/eco-communities. |
| Number of people involved | 2640-8170 | The report categorizes initiatives into 13 small (<10), 10 medium (10-100), 5 large (100-1000), and 2 very large (>1000). The low estimate assumes the lower boundary, the high estimate the upper boundary (except for very large). Additionally, we assume a minimum of 7 members per eco-villages/eco-communities. |
| Renewable capacities installed | 2.3-14.1 MW | 10 projects for renewable-based production of electricity and/or heat. Reported capacities in the database are 3.7 MW (thereof 0.51 MW solar PV, 2.3 MW wind, and 800 kW unspecified generation technology). The estimate also adds reported converted data for yearly generation if capacities are missing (corrected for technology specifications). As of 2018, all projects are under development which is why the lower estimate is zero. 2.3 MW are owned by Swiss initiatives. |
| Number of projects | 36 | Most projects are under development. Assuming 1 per initiative/ eco-village. |
| Total funds invested | N/A | Lack of information on planned projects and distribution of capacities to specific technologies. |

The following source was used in determining the aggregates for Norway: [92]

Norges Vassdrag og Energi Direktorat (NVE), "Descriptive Study of Local Energy Communities. Thema Consulting Group, Thema Report 2018-20" (NVE, 2018; <https://thema.no/wp-content/uploads/THEMA-Reort-2018-20-Local-Energy-Communities-Report-Final.pdf>).

## Poland (POL)

The most common legal forms in Poland are *Spółdzielnie* (cooperatives), including housing cooperatives. Furthermore, the informal concept of Energy Clusters has been established, but energy cooperatives do not have a legal form currently in Poland. The sector of energy communities is slowly growing, and Polish energy markets continue to open up for non-incumbents as laws and regulations fostering the national energy transition are being implemented, including EU Directives on energy communities. Currently, the overall assessment of the transposition of the EU Directives is that it is being implemented into national law, but so far suffers from several deficiencies such as limited citizen participation and absence of a designated authority to oversee that the definitions are lawfully enforced. Poland emphasizes the form of energy clusters where citizen-control is limited. In addition, there are 3 eco-villages and/or eco-communities in Poland.

Currently 12.2% of the country's final energy consumption is based on renewable energy [2]. Top-reported production technologies include solar PV (46%), geothermal heat (41%), biomass based heat (3%), and wind (2%). Other reported fields of engagement include retrofitting of houses, energy services (awareness raising, planning, consulting), and distribution (trade of electricity, ICT grid). Most energy clusters are in the planning phase. See Table S25 below for aggregate estimates of indicators for Poland.

**Table S25.** Aggregate estimates for Poland.

| **Indicator** | **Quantitative estimate** | **Notes** |
| --- | --- | --- |
| Number of initiatives | 121 | 45 housing cooperatives (25 in the database with more information) , 66 officially certified energy clusters (but only the 51 listed with additional information), 3 energy cooperatives, 4 other project collaborations, 3 eco-villages/eco-communities. |
| Number of people involved | 71720 | Data for 11 of 45 housing initiatives (71467 members), adding a minimum of 7 members for those not reporting, rounding: 71700.  Energy coops: data for two, round to account for third: 20 members.  Energy clusters: 68% provide information on members (rounded average 2018/2021): 15 members. We assume this average for those clusters not reporting. Rounding to 1000 members. We do not report the latter as citizen-leadership is not clear. |
| Renewable capacities installed | 154.5 MW | Installed capacities as reported by initiatives, the upper estimate includes capacities under development (2.5 MW wind, 4.5 MW biomass/biogas, 71 MW PV, 11 kW hydro, 63 MW geothermal). Two geothermal projects from 1997/1996, otherwise first production projects commissioned from 2013). Energy clusters: we only account for announced capacity by 10 pilot clusters of up to 2.5 MW (upper estimate). No correction for ownership due to lack of data. |
| Number of projects | 136 | 54 recorded production units from 39 initiatives. Assuming a project per initiative for the remaining (including project planning). |
| Total funds invested | 2.5 Million EUR | Based on PV capacities reported, excluding those from energy clusters as most of them are only announced plans without technical specifications and the control by citizens is limited. Solar capacities then amount to 2.1 MW. Assuming solar PV costs at average at 1200 EUR/kW, investment for solar sum up to about 2.5 mill. EUR. Governmental and regional funds are likely to cover the lion's share. Some initiatives report having received national and/or EU funding. |

The following sources were used in determining the aggregates for Poland: [93—96]

Krajowy Ośrodek Wsparcia Rolnictwa (KOWR), The National Support Centre for Agriculture (KOWR, 2021; [https://www.kowr.gov.pl/uploads/pliki/DI/Spóldzielnie%20enrgetyczne/Rejestr%20spółdzielni%20energetycznych/Wykaz%20spółdzielni%20energetycznych_2021.05.11.pdf](https://www.kowr.gov.pl/uploads/pliki/DI/Sp%C3%B3ldzielnie%20enrgetyczne/Rejestr%20sp%C3%B3%C5%82dzielni%20energetycznych/Wykaz%20sp%C3%B3%C5%82dzielni%20energetycznych_2021.05.11.pdf)).

Ministerstwo Aktywów Państwowych (Ministry of State Assets). *Krajowy plan na rzecz energii i klimatu na lata 2021–2030 (National plan for energy and climate for 2021–2030)* (2019).

Ministerial registry, (nd). <https://www.gov.pl/web/klimat/polityka-energetyczna-pol>.

J. Jazinski, M. Kozakiewicz, M. Soltysik, Determinants of Energy Cooperatives' Development in Rural Areas - Evidence from Poland. *Energies*. **14/2**, 319 (2021). <https://doi.org/10.3390/en14020319>.

## Portugal (PRT)

The most common legal form in Portugal is cooperative (*cooperativa*). In recent years, only one energy cooperative started in 2013 and grew considerably with the development of a number of renewable energy communities throughout the country. In our inventory, we report on 8 initiatives. In the 1930s, 7 cooperatives were founded for the operation of electricity grids in rural areas. Since 2013, the big-sized cooperative *Coopernico* has developed 29 projects. In addition, 26 eco-villages, eco-cities, eco-communities, and/or transition towns exist in Portugal.

In the past, the framework conditions favored large commercial actors in the installation of renewable energies. Nonetheless, one cooperative seems to have found a niche in supporting energy communities, resulting in the installation of 29 PV production units so far. The fields of engagement include solar PV, hydropower, and consultancy. Existing cooperatives focus on the development of renewable energy communities throughout the country. Some existing hydropower plants were bought by a cooperative from Spain.

It remains to be seen how the policy changes from 2021 to allow self-consumption of renewable energy on a collective level and by renewable energy communities will help encourage citizens to engage in production and self-consumption of renewable energy sources. A good hint may be the *Coopernico* cooperative which helped a fast-growing number of energy communities to develop. By early 2022 both definitions of REC and CEC were introduced into national law. However, there are some challenges to the transposition of the EU directives into national law, with some of the EU criteria being misinterpreted, consequently having citizens risk becoming passive members rather than active members.

Currently 28.2% of the country's final energy consumption is based on renewable energy [2]. Top-reported production technologies used by initiatives are hydro (57%) and solar PV (43%). Other fields of engagement include electricity trade and grid operation. See Table S26 below for aggregate estimates of indicators for Portugal.

**Table S26.** Aggregate estimates for Portugal.

| **Indicator** | **Quantitative estimate** | **Notes** |
| --- | --- | --- |
| Number of initiatives | 37 | Only one of the 9 cooperative initiatives is a recent one, 2 associations. Includes 26 eco-villages/eco-communities. |
| Number of people involved | 2400-45000 | The seven grid operating cooperatives have limited information about their membership base but list 41500 customers. The minimum number of members is 3, but web pages list on average 6 members of the board. We assume 10 members per historical coops. The biggest and most recent initiative refers to 2240 members and 1800 customers in 2020. Additionally, a minimum of 7 members per eco-villages/eco-communities are assumed. Rounding. The upper range includes customers. |
| Renewable capacities installed | 4.4 MW | 2.2 MWp Solar PV, 6.5 MW hydropower installed capacity. Correcting for ownership leads to the estimates (e.g., hydropower with 25%). |
| Number of projects | 69 | Production units: 32 small and medium-size solar PV rooftop installations (from one cooperative alone), 4 hydropower plants. Assuming 1 project per eco-village and 1 for each remaining 7 initiatives with no production info. |
| Total funds invested | 17.93 Million EUR | Accounting for the projects of the biggest and most recent initiative alone, the sum of investments is 1.77 mill. EUR. Three initiatives report investments for 2018 of 16.102 mill. EUR. Note that Copernico, which is the largest cooperative, manages about 240 MW capacities for their owners. |

The following sources were used in determining the aggregates for Portugal: [30, 59, 97—99]

D. Frieden, A. Tuerk, C. Neumann, S. d'Herbemont, J. Roberts, REScoop.eu, "Collective Self-Consumption and Energy Communities: Trends and Challenges in the Transposition of the EU Framework. Working Paper" (REScoop.eu., 2020; <https://www.rescoop.eu/uploads/rescoop/downloads/Collective-self-consumption-and-energy-communities.-Trends-and-challenges-in-the-transposition-of-the-EU-framework.pdf>).

I. Douvitsa, The New Law on Energy Communities in Greece. *Cooperativismo e Economía Social*. **40** (2018). <https://doi.org/10.35869/ces.v0i40.1385>.

H. Algarvio, The Role of Local Citizen Energy Communities in the Road to Carbon-Neutral Power Systems: Outcomes from a Case Study in Portugal. *Smart Cities.* **4/2**, 840-863 (2021). <https://www.mdpi.com/2624-6511/4/2/43/htm>.

Coopérnico, *Energia verde, sustentabilidade e cidadania* (2021). <https://www.coopernico.org/>.

S. Sareen, "Scalar Biases in Solar Photovoltaic Uptake, Socio-Materiality, Regulatory Inertia and Politics" in *Dilemmas of Energy Transitions in the Global South: Balancing Urgency and Justice*, A. Kumar, J. Höffken, A. Pols, Eds. (London: Routledge, 2021). <https://doi.org/10.4324/9780367486457>.

## Romania (ROU)

The most common legal form in Romania is *Societate Cooperativă Europeană* (European cooperative society). However, legal and financial barriers for citizens to engage are high. Strong centralization of legislative and executive powers makes it difficult for local parties to participate. This is reflected in the ongoing implementation of the EU Directives into national law, with low transposition and copy-paste tendencies, rather than adaptation of the definitions into national law. Citizen participation is very limited in the present legislation.

Still, one recently founded initiative engages in solar PV and electricity trade. Cebotari [100] collected data between 2015-2016 and indicates two other community-owned renewable energy initiatives. However, as discussed in the paper, the community benefit in both is assessed to be small and decision-making lies with the local government, which is why we did not include the candidates in the inventory. In addition, 4 eco-villages/eco-communities exist in Romania.

Currently 23.5% of the country's final energy consumption is based on renewable energy [2]. See Table S27 below for aggregate estimates of indicators for Romania.

**Table S27.** Aggregate estimates for Romania.

| **Indicator** | **Quantitative estimate** | **Notes** |
| --- | --- | --- |
| Number of initiatives | 5 | A recently founded energy cooperative plus 4 eco-villages/eco-communities. |
| Number of people involved | 750 | The number has been growing since 2019 from 15 initial members to 704 in 2021. We add 7 members as a minimum per eco-village. Rounding. |
| Renewable capacities installed | 5465 kWp | 1 solar PV production unit, ownership assumed 100%. |
| Number of projects | 5 | Assumes 1 per initiative. |
| Total funds invested | 0.4- 4.5 Million EUR | 0.4 mill. EUR invested for acquisition of electricity trade supplier. Additional funds for solar PV installation are estimated assuming 750 EUR/kWp *(78)*. The low estimate assumed 0% ownership, the high 100%. |

The following sources were used in determining the aggregates for Romania: [64, 100]

International Energy Agency (IEA), "National Survey Report of PV Power Applications in Italy 2020" (IEA Photovoltaic Power Systems Programme, 2020; <https://iea-pvps.org/wp-content/uploads/2021/11/NSR_Italy_2020.pdf>).

S. Cebotari, Against All Odds: Community-Owned Renewable Energy Projects in North-West Romania. *ACME: An International Journal for Critical Geographies*. **18/2**, 513-528 (2019). <https://acme-journal.org/index.php/acme/article/view/1556>.

## Slovakia (SVK)

Community-based energy initiatives are only in their infancy in Slovakia [101, 102]. The most common legal form identified in Slovakia is *Družstvo* (cooperative). The vast majority of these initiatives are agricultural cooperatives that recently also engage in biogas production, biofuel-based electricity production, and/or the provision of consultancy services for energy installations. In addition, 2 eco-villages exist in Slovakia. Implementation of EU Directives into national law is currently ongoing. The national law will put forward concrete measures and reduce administrative barriers. No update on the status of the implementation of the directives is provided in the REScoop policy tracker.

Currently 17.6% of the country's final energy consumption is based on renewable energy [2]. Biomass is by far the most deployed production technology. Other reported fields of engagement include electricity generation from PV and hydropower, energy consulting services, and energy efficiency measures. See Table S28 below for aggregate estimates of indicators for Slovakia.

**Table S28.** Aggregate estimates for Slovakia.

| **Indicator** | **Quantitative estimate** | **Notes** |
| --- | --- | --- |
| Number of initiatives | 25 | Included all initiatives with Družstvo in its name and as legal form, 2 eco-villages. |
| Number of people involved | 175 | Data is not available, assuming at least 7 people per initiative. |
| Renewable capacities installed | 14.5 MW | Reported are 11 units with biomass-based heat production (6.120 MW), 6 units with biomass-based electricity production (5.9 MW), 1 biomass-based cogeneration plant with 0.25 MW heat and 0.19 MW electricity production, 3 solar PV units (1.187 MW), and 2 hydropower units (0.860 MW). |
| Number of projects | 56 | As reported and assuming at least one project/initiative for those not reporting. |
| Total funds invested | 26.374 Million EUR | Estimate based on capacities: Solar PV - 1190 kW*3350 EUR [103]. Heat - 700 kW*3300 EUR. Biomass-based electricity - 6087 kW*3300 EUR. Rounding. No attempt is made to estimate investments for 2 hydropower plants, whose production started after 2000. |

The following sources were used in determining the aggregates for Slovakia: [101—104]

Community Power, Slovakia (nd). <https://www.communitypower.eu/en/slovakia.html>.

R. Leal-Arcas, B. Burstein, M. Mattera, Electrifying the Energy Sector: The Case of Slovakia and the Czech Republic. *Kentucky Journal of Equine, Agriculture, & Natural Resources Law* (Queen Mary School of Law, Legal Studies Research Paper no. 329/2020). **13/1**, 1-83 (2020). <https://www.researchgate.net/publication/339200302>.

International Renewable Energy Agency (IRENA), "Renewable Power Generation Costs in 2020" (IRENA, Abu Dhabi, 2021; <https://www.irena.org/-/media/Files/IRENA/Agency/Publication/2021/Jun/IRENA_Power_Generation_Costs_2020.pdf>).

European Commission, "Commission Staff Working Document, Assessment of the Final National Energy and Climate Plan of Slovakia. National Energy and Climate Plans (NECP). SWD (2020), 924 Final" (European Commission, 2020; <https://ec.europa.eu/energy/sites/default/files/documents/staff_working_document_assessment_necp_slovakia_en.pdf>).

## Slovenia (SVN)

According to Palm *et al.* (2020) [105], citizen-led energy initiatives are still in their infancy in Slovenia. When compared with this and other previous studies, we find that we are likely to capture existing initiatives. Palm [105] identified 2 existing initiatives, and the Compile project [106] identified 1 additional initiative. In our inventory, we report on 8 initiatives. In addition, Slovenia has 3 eco-villages. We only report known information without attempting to propose estimates, except for the number of projects where we assume at least 1 project per initiative. The database provides the links to the reports.

The most common legal form in Slovenia is a Cooperative Limited Liability (*Zadruga z omejeno odgovornostjo*). Implementation of EU directives are ongoing, where simplification of administrative procedures and enabling a framework for renewable energy communities are important focus points for national legislation. No update from the REScoop policy tracker is provided. Currently 20.9% of the country's final energy consumption is based on renewable energy [2]. Reported production technologies include both solar PV and onshore wind. See Table S29 below for aggregate estimates of indicators for Slovenia.

**Table S29.** Aggregate estimates for Slovenia.

| **Indicator** | **Quantitative estimate** | **Notes** |
| --- | --- | --- |
| Number of initiatives | 11 | Cooperative limited liabilities, 3 eco-villages. |
| Number of people involved | 77 | Assuming at least 7 members per initiative. |
| Renewable capacities installed | 294 kWp | 5 reported solar PVproduction units, which range between 42-102 kWp. |
| Number of projects | 12 | 5 projects, 2 of which belong to the same initiative, plus 3 eco-villages. Assuming at least 1 project initiative leads to the final estimate. |
| Total funds invested | 0.252-0.454  Million EUR | Based on 9 projects with 300 kWp and 4 x 60 kWp (based on the assumption of additional projects and average of solar PV capacities). Assuming 840 EUR/kWp (reported from 2019 [103]). |

The following sources were used in determining the aggregates for Slovenia: [93, 103, 105—107]

Krajowy Ośrodek Wsparcia Rolnictwa (KOWR), The National Support Centre for Agriculture (KOWR, 2021; [https://www.kowr.gov.pl/uploads/pliki/DI/Spóldzielnie%20enrgetyczne/Rejestr%20spółdzielni%20energetycznych/Wykaz%20spółdzielni%20energetycznych_2021.05.11.pdf](https://www.kowr.gov.pl/uploads/pliki/DI/Sp%C3%B3ldzielnie%20enrgetyczne/Rejestr%20sp%C3%B3%C5%82dzielni%20energetycznych/Wykaz%20sp%C3%B3%C5%82dzielni%20energetycznych_2021.05.11.pdf)).

International Renewable Energy Agency (IRENA), "Renewable Power Generation Costs in 2020" (IRENA, Abu Dhabi, 2021; <https://www.irena.org/-/media/Files/IRENA/Agency/Publication/2021/Jun/IRENA_Power_Generation_Costs_2020.pdf>).

J. Palm, K. Reindl, S. Sommer, "Deliverable D3, Description of Polycentric Settings in the Partner Countries. New Clean Energy Communities in a Changing European Energy System (NEWCOMERS) Project, Deliverable D3" (NEWCOMERS, 2020; <https://www.researchgate.net/publication/342336463_New_Clean_Energy_Communities_in_a_Changing_European_Energy_System_NEWCOMERS_Deliverable_D3_1_Description_of_polycentric_settings_in_the_partner_countries/link/5f16cb30a6fdcc9626a437e1/download>).

Compile Project, "Installation and Connection of PV in Luče, Slovenia" (COMPILE Project, 2019; <https://www.compile-project.eu/news/installation-and-connection-of-pv-in-luce-slovenia/>).

European Commission, "Commission Staff Working Document, Assessment of the Final National Energy and Climate Plan of Slovenia. SWD (2020) 923 Final" (European Commission, 2020; <https://ec.europa.eu/energy/sites/default/files/documents/staff_working_document_assessment_necp_slovenia_en.pdf>).

## Spain (ESP)

The most common legal form in Spain is *Sociedad cooperativa* (cooperative). The implementation of the REC definition is complete with the transposition of the CEC definition expected to be implemented in 2022. Energy communities are currently emerging on a monthly basis. Most of them start with small PV systems supplying energy for consumption in a couple of neighboring households. Having started to operate under the legal umbrella of the municipality, the energy communities will decide later to opt for a cooperative or an association model. Overall, citizen participation is an issue of discussion, but the latest regulations eased individual and collective self-consumption. Self-consumption was previously restricted to only generation facilities and consumers located in the same dwelling. Now, participation in self-consumption systems extends to consumers within a 500-meter radius around the installation, now allowing community members, groups of apartment owners, entire neighborhoods, or industrial estates to participate together. This has resulted in a recent surge in the foundations of energy communities.

As indicated above, the situation of citizen-led energy initiatives in Spain is very dynamic at the moment. In addition to historical electric cooperatives who also engage in renewable energy projects, many new initiatives have been created in the form of a cooperative over the last 5 years. An additional 120 eco-villages, eco-cities, eco-communities, and 1 transition town exist in Spain.

Currently 17.3% of the country's final energy consumption is based on renewable energy [2]. Solar PV (46%) and onshore wind (49%) are by far the most frequent production technology utilized by initiatives. Solar PV projects are realized especially among energy communities that are mostly active in neighborhoods. Note that the ownership of grid infrastructure from initiatives is substantial. Other reported fields of engagement include electricity trading, grid operation, consultancy, supporting self-consumption, and awareness raising. Cooperatives are often engaged in trading electricity and operating local electricity grids. See Table S30 below for aggregate estimates of indicators for Spain.

**Table S30.** Aggregate estimates for Spain.

| **Indicator** | **Quantitative estimate** | **Notes** |
| --- | --- | --- |
| Number of initiatives | 358 | 67 cooperatives, 170 energy communities (ECs) (of which 43 active, 127 in foundation/feasibility study), plus 120 eco-villages/transition towns. |
| Number of people involved | 185440 | Member data for 32 initiatives that are not EC between 2018-2022: 141500 members (includes Somergia with 81500 members), we estimate for the remaining 35 coops not reporting their membership based on the mean (1027, excluding the two largest cooperatives from the calculation): 35950 members. Total: ~177450. EC**:** Based on the average of 29 ECs with a given amount of installed PV capacity who supply ~1300 households: 11 active ECs with unknown amount of installed capacity: ~550 households supplied = members of EC. 35 ECs developing installations:~1800 households supplied = members of EC, 69 ECs in foundation ~3450 households to be supplied = members of EC. Total estimation for ECs from electricity supplied: 7100 members. Additionally adding a minimum of 7 members per eco-villages/transition towns. Rounding. |
| Renewable capacities installed | 101- 207 MW | Ownership weighted capacity assuming 0% (100%) for unknown ownership: Solar PV/CSP capacities: 50 MW (73 MW). Biomass-based heat/co-generation: 1 MW (1 MW). Hydropower: 5.4 MW (6.7 MW). Wind: 79 MW. 25 units planned/under construction with an additional 48 MW are added to the higher estimate. 88 ECs do not report. Note that the ownership of grid infrastructure is substantial but not quantified here. Note that 83 MW are installed in Spain, which are owned by Swiss initiatives. |
| Number of projects | 370 | Assumption of at least one project per initiative, accounting for 158 production units, 22 other projects and offers made by initiatives. |
| Total funds invested | 65.8-113.8 Million EUR | Financial investments for 23 production units reported sum up to 45.870 mill. EUR. Financial investments for solar capacities (x 900 EUR/kWp for installations after 2018 and 2000 EUR/kWp for the few installations before): 17.521 mill. EUR. One newer hydropower plant is accounted for at costs of 2400 EUR/kW: 2.4 mill. EUR. No ground for estimation of other RE capacity investments. We add the planned wind capacity investments to the higher estimate, assuming 1 mill. EUR/MW. Note that the production units owned by Swiss initiatives are accounted for in CHE. |

The following sources were used in determining the aggregates for Spain: [108—110]

International Energy Agency (IEA), "National Survey Report of PV Power Applications in Spain, 2020" (IEA Photovoltaic Power Systems Programme, 2020; <https://iea-pvps.org/wp-content/uploads/2021/09/NSR_Spain_2020_b.pdf>).

I. Heras-Saizarbitoria, L. Sáez, E. Allur, J. Morandeira, The Emergence of Renewable Energy Cooperatives in Spain: A Review. *Renewable and Sustainable Energy Reviews.* **94**, 1036–1043 (2018). <https://doi.org//10.1016/j.rser.2018.06.049>.

C. Gallego-Castillo, M. Heleno, M. Victoria, Self-Consumption for Energy Communities in Spain: A Regional Analysis Under the New Legal Framework. *Energy Policy*. **150**, 112144 (2021). <https://doi.org/10.1016/j.enpol.2021.112144>.

## Sweden (SWE)

Wind cooperatives are the dominant form of citizen-led initiatives in Sweden. The country experienced a boom in wind cooperatives between 1990-2010 due to favorable legislation (e.g., high CO_2_ tax, RE subsidies). Changes in legislation halted the establishment of new wind cooperatives. Since 2010 there has been a decrease in initiatives, but with some recent growth in solar PV initiatives [111]. The most common legal forms in Sweden are *Ekonomiska förening* (economic association), *Samfällighetsförening* (joint-ownership association), *Bostadsförening* (housing association), *Bostadsrättsförening* (tenant owner's association), *Ideell förening* (non-profit organization), and *Enkeltbolag* (regulated partnership between two partners). Most economic associations are wind cooperatives, while housing cooperatives often engage in electricity trading (consumption) and rooftop solar PV installations. Additionally, 32 eco-villages exist in Sweden.

The implementation of the EU directives are ongoing. The Swedish Energy Regulator has proposed recommendations for how to transpose a new law on RECs and CECs, but it has yet to be formally introduced to the government. The new law proposes one concept, energy communities, and largely concurs with the EU directive criteria.

Currently 52.9% of the country's final energy consumption is based on renewable energy [2]. The top-reported production technologies used by initiatives include wind (94%), solar PV (3%), hydro (2%), as well as heat, biofuels and geothermal accounting for 1% jointly. Other reported fields of engagement include electricity trade, grid operation, consulting, mobility, awareness raising, and R&D activities. See Table S31 below for aggregate estimates of indicators for Sweden.

**Table S31.** Aggregate estimates for Sweden.

| **Indicator** | **Quantitative estimate** | **Notes** |
| --- | --- | --- |
| Number of initiatives | 336 | 336 initiatives. We count a total of 32 eco-villages, but not all provide further information. We report 12 stock companies, which we count into initiatives following Hewitt *et al.* (2019) [115]. In addition, 61 initiatives are active in the energy sector, but concrete information about their engagement in renewable energy specifically is missing. 94 initiatives that are mostly engaging in non-energy related activities, mainly housing, are also counted towards the number of initiatives as we can clearly identify projects related to the low carbon energy transition. |
| Number of people involved | 124500 | 94 initiatives report on membership (2007-2020). Two large initiatives alone have 82000 members, but most have below 300 members. We take the most recent information on members to report the number of people involved over the period, rounding. We assume 10 members for initiatives not reporting. Rounding. |
| Renewable capacities installed | 170.31- 264.96 MW | 140 initiatives report a total 324.885 MW capacity for 276 of 310 production units/equipment, ownership information is provided for 226 of them. For those where ownership information is provided, capacities are adjusted accordingly. For those not reporting on ownership, we provide a low and a high estimate assuming 0% and 100% ownership, respectively. Thus, wind (low: 162.73 MW, high: 242.55 MW), solar PV (low: 1.25 MWp, high: 7.23 MWp), hydropower (6.33 MW - ownership data fully accounted for), biofuel/biomass/heat (high: 2.8 MW). |
| Number of projects | 375 | 375 energy-related activities are recorded in the database, 310 production units/equipment-based units are registered. |
| Total funds invested | 229.5-369.3 Million EUR | Based on installation cost per kW installed capacity. Wind (228-340 mill EUR): Classification into two time periods (<=2002, >2002), using installation costs for the year 2000 and 2010 for the first and second category, respect.. Choice of years due to clear peaks of newly installed units in the years around 2000 and 2010. Cost data taken from Duffy [45] and IRENA [103]. Lower estimate assumes 0% ownership for those not reporting; upper estimate assumes 100%. Solar PV (1.5-6.5 mill. EUR): Most solar PV installations have not reported a year of installation. Those with reported information on year of installation were all installed after 2014. Those with no information were consequently assumed to have been installed after 2014. An average system price for Swedish PV installation for 2014-2020 was used for the investment calculation [2]. The price for roof mounted installations of a size of 100kW was used in all cases except one large ground mounted facility with almost 6 MW. Here, the price for ground-mounted units larger than 500 kW was used. Detailed information on share size and number of shares is available for 8 initiatives. These sum up to a combined investment of 22.8 mill. EUR. The 10 Year average exchange rate for SEK to EUR was used to convert final investment values, as baseline information relates to this timeframe. We report only under the high estimate. |

The following sources were used in determining the aggregates for Sweden: [45, 103, 111—115]

A. Duffy, M. Hand, R. Wiser, Land-Based Wind Energy Cost Trends in Germany, Denmark, Ireland, Norway, Sweden and the United States. *Applied Energy*. **277**, 114777 (2020). <https://doi.org/10.1016/j.apenergy.2020.114777>.

International Renewable Energy Agency (IRENA), "Renewable Power Generation Costs in 2020" (IRENA, Abu Dhabi, 2021; <https://www.irena.org/-/media/Files/IRENA/Agency/Publication/2021/Jun/IRENA_Power_Generation_Costs_2020.pdf>).

D. Magnusson, J. Palm, Come Together—The Development of Swedish Energy Communities. *Sustainability*. **11/4**, 1056 (2019). <https://doi.org/10.3390/su11041056>.

International Energy Agency (IEA), "National Survey Report of PV Power Applications in Sweden" (IEA, 2020; <https://iea-pvps.org/wp-content/uploads/2021/10/National-Survey-Report-of-PV-Power-Applications-in-Sweden-2020.pdf>).

E. Lantz, M. Hand, R. Wiser, "The past and future cost of wind energy" paper presented at the World Renewable Energy Forum 2012, Denver, CO, 13-17 May 2021.

T. Wizelius, "Community Wind in Sweden" in *Handbuch Energiewende und Partizipation*, Holstenkamp, L. & Radtke, J., Eds. (Springer VS: Wiesbaden, 1047-1059, 2018). <https://doi.org/10.1007/978-3-658-09416-4_62>.

R. Hewitt, N. Bradley, A. Baggio Compagnucci, C. Barlagne, A. Ceglarz, R. Cremades, M. McKeen, I. Otto, B. Slee, Social Innovation in Community Energy in Europe: A Review of the Evidence. *Frontiers in Energy Research*. **7/31** (2019). <https://doi.org/10.3389/fenrg.2019.00031>.

## Switzerland (CHE)

The most common legal forms in Switzerland are *Genossenschaft*, *Société coopérative*, and *Società cooperativa* (all for cooperatives). Energy cooperatives have a long tradition in Switzerland. The first cooperatives were organized at the end of the 19th century as part of the electrification of rural areas. Many of them still exist today, yet their number is declining. At the beginning of the 21st century, a variety of new initiatives were established.

Currently, 24.8% of the country's final energy consumption is based on renewable energy [2]. The top production technologies reported by initiatives include hydro (57%), solar PV (33%), heat/wood-based heat (6%), and wind (4%). Other fields of engagement include distribution, biogas, and consulting services. Notably, three larger cooperatives are among the country's largest utilities and account for the majority of installed capacity. Their units, larger wind and solar farms, are mostly located outside of Switzerland. Since 2016, and initiated by the initiative EWG, new cooperatives have been founded for joint purchasing and do-it-yourself collaboration. The latter is supporting the installation of small-scale PV systems on private homes and the owner commits a specific amount of time to support the next installation on a private home with her new gained expertise. It helps to save costs. Additionally, four eco-villages exist in Switzerland. Notably, two cooperatives operate as large-scale electricity utilities with 67 000 members accounting for a large fraction of installed capacity and having an international portfolio of solar PV, CSP, and wind projects. See Table S32 below for aggregate estimates of indicators for Switzerland.

**Table S32.** Aggregate estimates for Switzerland.

| **Indicators** | **Quantitative estimate** | **Notes** |
| --- | --- | --- |
| Number of initiatives | 297 | All of them are cooperatives mainly active in the energy sector (116 from before 1990). 4 eco-villages. |
| Number of people involved | 84470 | 35 initiatives report data on membership. The typical size is between 50-150 members. The total is 71569 (the two largest have 55000 and 12 000 members alone). Most of the 55 initiatives active in heat production do not report members and these initiatives tend to be of smaller size. At the same time, grid-operating Elektras are typically much larger. Thus, we assume on average 50 members for those initiatives not reporting, adding 12900. Rounding. |
| Renewable capacities installed | 50- 94 MW | Details on 417 production units are reported by 78 initiatives, 47 reveal information on ownership shares. Not correcting for ownership shares, the total amount of capacities sums up to 858 MW.  High (low) estimates for ownership-corrected capacities amount to 4.18 (0) MW for wind, 54 (50) MW for hydropower, 31 (0) MW for solar PV, and 5.4 (0) MW for heat/wood-based heat.  Notably, a substantial share of capacities (193-196 MW) owned by Swiss initiatives is located outside of Switzerland. |
| Number of projects | 2580 | 230 initiatives report information on activities (121 operate grids, 55 engage in heat production and grid operation, 103 produce electricity (⅓ of the initiatives), 26 offer consulting services and 9 support planning and construction of renewable generation which led to ~2000 projects. 417 of these are renewable capacity production units, which are registered. We record at least ~100 activities related to energy services. Together with the ~2000 projects, we arrive at 2516. In addition, we assume for those initiatives without further information that they have at least 1 project (+63 projects). We assume 1 per eco-village. Rounding. |
| Total funds invested | 344.4 Million EUR | Financial investment data are available for 66 production units, amounting to 13.21 mill. EUR (corrected for exchange rates). Assuming mean installation cost of 2500 CHF per kWp for PV systems (own calculation; see also [130]), the total PV investments based on individual unit entries is estimated at 129.25 mill. EUR via extrapolation. Investments in wind power are estimated at 196 mill. EUR, based on data for the capacity of production units (assuming 1230 EUR/kW). Furthermore, an additional 10 initiatives report on annual investments in general, summing to 5.98 mill. EUR. Note, that for heat, CSP, and hydro power it was not possible to estimate investments given heterogeneity of environments and infrastructure. Investment data is corrected for ownership information, that is, only the amount of capacity owned by the initiative is utilized for financial estimates. |

The following source was used in determining the aggregates for Switzerland: [116]

International Energy Agency (IEA). *National survey report of PV power applications in Switzerland, 2019*. IEA photovoltaic power systems programme. <https://iea-pvps.org/wp-content/uploads/2020/09/NSR_Switzerland_2019_v2.pdf> (2019).

# References

1. RESCOOP, REScoop policy tracker. (2022). <https://www.rescoop.eu/policy#transposition-tracker>.
2. International Energy Agency (IEA), Renewables page (2022). https://www.iea.org/fuels-and-technologies/renewables.
3. Global Ecovillage Network (GEN), Ecovillages Projects page (2022). <https://ecovillage.org/projects/>.
4. Transition Network, website homepage (2022). <https://transitionnetwork.org/>.
5. A. Wierling, V.J. Schwanitz, J.P. Zeiß, C. Bout, C. Candelise, W. Gilcrease, J.S. Gregg, Statistical Evidence on the Role of Energy Cooperatives for the Energy Transition in European Countries. *Sustainability*. **10**, 3339 (2018). <https://doi.org/10.3390/su10093339>.
6. AGRAR PLUS, "Heizwerk in Niederöstereich - Broschüre zu Heizwerken und erneuerbarer Energie in NÖ, Report 150" (Agrar Plus, nd; <https://agrarplus.at/folder-broschueren.html?file=files/agrarplus-inhalte/folder_broschueren/folder_150_heizwerk.pdf&cid=127>).
7. AGRAR PLUS, "Erneuerbare Energie in Niederösterreich, Report 200" (Agrar Plus, nd; <https://agrarplus.at/folder-broschueren.html?file=files/agrarplus-inhalte/folder_broschueren/folder200_heizwerk.pdf&cid=126>).
8. FirmenABC, Web service mirroring the Austrian Business Registry (2022). <https://www.firmenabc.at>.
9. Open Infra Map, Map of Austrian Power Plants (2022). <https://openinframap.org/stats/area/Austria/plants>.
10. M. Seiwald, The (Up) Scaling of Renewable Energy Technologies: Experiences from the Austrian Biomass District Heating Niche. *Moravian Geographical Reports*. **22/2**, 44-54 (2014). <https://doi.org/10.2478/mgr-2014-0011>.
11. T. Bauwens, B. Gotchev, L. Holstenkamp, What Drives the Development of Community Energy in Europe? The Case of Wind Power Cooperatives. *Energy Research & Social Science.* **13**, 136-147 (2016). <https://doi.org/10.1016/j.erss.2015.12.016>.
12. D. Holemans, K. Van de Velde, "Citizens Energy: Making Energy Democracy Happen" (Green European Foundation & OIKOS Report, 2019; <https://gef.eu/wp-content/uploads/2019/01/GEF_Oikos_Citizens-Energy_Print.pdf>).
13. B. Wilken, International Energy Agency (IEA), "National Survey Report of PV Power Applications in Belgium, 2018" (IEA Photovoltaic Power Systems Programme, 2018; <https://iea-pvps.org/wp-content/uploads/2020/01/NSR_Belgium_2018.pdf>).
14. International Energy Agency (IEA), "National Survey Report of PV Power Applications in France, 2019" (IEA Photovoltaic Power Systems Programme, 2019; <https://iea-pvps.org/wp-content/uploads/2020/09/NSR_France_2019.pdf>).
15. G. Limpens, H. Jeanmart, F. Maréchal, Belgian Energy Transition: What Are the Options? *Energies.* **13/1**, 261 (2020). <https://doi.org/10.3390/en13010261>.
16. S. Oberthur, Ó. Söebech, H. Derde, K. Jackers, J. Vanhoenacker, T. Vermeir, W. Vandorpe, D. Haverbeke, "Legislative Options and Obstacles for Energy Communities in Belgium: Summary of Key Issues Identified & Recommendations" (ROLECS Project, Task 2.3.1, 2020; <https://cris.vub.be/ws/portalfiles/portal/55931943/Energy_communities_in_Belgium_legal_analysis_Oct_2020.pdf>).
17. M. Vladimirov, A. Georgiev, S. Kolarova, "Development of Small-Scale Renewable Energy Sources in Bulgaria: Legislative and Administrative Challenges" (Center for the Study of Democracy, 2018; <https://csd.bg/publications/publication/development-of-small-scale-renewable-energy-sources-in-bulgaria-legislative-and-administrative-chal/>).
18. I. Todorović, "Bulgarian Municipalities Advised on Possibilities for Energy Communities" (Balkan Green Energy News, 2020; <https://balkangreenenergynews.com/bulgarian-municipalities-advised-on-possibilities-for-energy-communities/>).
19. I. Todorović, "Niš is Empowering Citizens within its Energy Transition" (Balkan Green Energy News, 2021; <https://balkangreenenergynews.com/nis-is-empowering-citizens-within-its-energy-transition/>).
20. Ministry of Energy and Ministry of the Environment and Water, "Integrated Energy and Climate Plan of the Republic of Bulgaria 2021-2030" (Republic of Bulgaria, 2019; <https://ec.europa.eu/energy/sites/default/files/documents/bg_final_necp_main_en.pdf>).
21. Izgrei, All Electricity Suppliers in One Place (2022). <https://www.izgrei.bg/>.
22. European Commission, "Commission Staff Working Document, Assessment of the Final National Energy and Climate Plan of Bulgaria, SWD (2020) 901 Final" (European Commission, 2020; <https://ec.europa.eu/energy/sites/default/files/documents/staff_working_document_assessment_necp_bulgaria_en.pdf>).
23. J. Eichermüller, M. Furlan, K. Habersbrunner, Z. Kordić, "Energy Cooperatives - Comparative Analysis in Eastern Partnership Countries and Western Balkans" (Women Engage for a Common Future, Zelena Energetska Zadruga (ZEZ), 2018; <http://www.wecf.org/wp-content/uploads/2018/06/EnergyCoops_LongOnline.pdf>).
24. E. Jerkic, "Energy Coops in Croatia" (Zelena Energetska Zadruga, 2016; <https://www.irena.org/-/media/Files/>[IRENA/Agency/Events/2016/Jan/18/ZEZ-Energy-coops-in-Croatia.pdf?la=en&hash=E72E78396642F36E972B9EE840CD09745A419746](https://www.irena.org/-/media/Files/IRENA/Agency/Events/2016/Jan/18/ZEZ-Energy-coops-in-Croatia.pdf?la=en&hash=E72E78396642F36E972B9EE840CD09745A419746)).
25. M. Kirac, M. Furlan, R. Pašičko, "Green Power to the People – Croatian Perspective on Community Energy" (Heinrich Böll Stiftung Belgrade, 2018;

<https://rs.boell.org/en/2020/04/10/green-power-people-croatian-perspective-community-energy>).

1. Cyprus Energy Authority (CEA), Energy Communities (2021). [https://www.cea.org.cy/en/energeiakes-koinotites](https://www.cea.org.cy/en/energeiakes-koinotites/).
2. Electricity Authority of Cyprus (EAC), Non Regulated Activities (2021). <https://www.eac.com.cy/EN/NonRegulatedActivities/about/Pages/default.aspx>.
3. European Commission, "Commission Staff Working Document, Assessment of the Final National Energy and Climate Plan of Cyprus. SWD (2020) 912 Final" (European Commission, 2020; <https://ec.europa.eu/energy/sites/default/files/documents/staff_working_document_assessment_necp_cyprus_en.pdf>).
4. European Commission, "Commission Staff Working Document, Assessment of the Final National Energy and Climate Plan of Czechia. SWD (2020) 902 Final" (European Commission, 2020; <https://ec.europa.eu/energy/sites/default/files/documents/staff_working_document_assessment_necp_czechia_en.pdf>).
5. D. Frieden, A. Tuerk, C. Neumann, S. d'Herbemont, J. Roberts, REScoop.eu, "Collective Self-Consumption and Energy Communities: Trends and Challenges in the Transposition of the EU Framework. Working Paper" (REScoop.eu., 2020; <https://www.rescoop.eu/uploads/rescoop/downloads/Collective-self-consumption-and-energy-communities.-Trends-and-challenges-in-the-transposition-of-the-EU-framework.pdf>).
6. V. Malý, M. Šafařík, R. Matoušek, "Consumer (Co-)Ownership in Renewables in the Czech Republic" in *Energy Transition Financing Consumer Co-Ownership in Renewables*, J. Lowitsch Ed. (Palgrave Macmillan: Cham, 2019), pp. 201-222.<https://doi.org/10.1007/978-3-319-93518-8>.
7. L. Gorroño-Albizu, K. Sperling, S. Djørup, The Past, Present and Uncertain Future of Community Energy in Denmark: Critically Reviewing and Conceptualising Citizen Ownership. *Energy Research & Social Science*. **57**, 101231 (2019). <https://doi.org/10.1016/j.erss.2019.101231>.
8. F. Mey, M. Diesendorf, Who Owns an Energy Transition? Strategic Action Fields and Community Wind Energy in Denmark. *Energy Research & Social Science*. **35**, 108–117 (2018). <https://doi.org/10.1016/j.erss.2017.10.044>.
9. Energiatalgud, Energiaühistud (2021). <https://energiatalgud.ee/Energia%C3%BChistud?category=736>.
10. European Commission, "Commission Staff Working Document, Assessment of the Final National Energy and Climate Plan of Estonia. SWD (2020) 905 Final" (European Commission, 2020; <https://ec.europa.eu/energy/sites/default/files/documents/staff_working_document_assessment_necp_estonia_en.pdf>).
11. E. Risthein, T. Vaimann, *Eesti energeetika 100 aastat*. Post factum (2018).
12. Chambers & Partners, Finland: An Introduction to Banking & Finance (2021). <https://chambers.com/content/item/3909>.
13. J. Korteniemi, "Mankala Principle: A Concept to Finance Large Clean Energy Investments in Finland" (Finland Ministry of Economic Affairs and Employment, 2018; <https://www.ifnec.org/ifnec/upload/docs/application/pdf/2018-11/s3_3_fin_korteniemi.pdf>).
14. Eurostat, Electricity Prices for Household Consumers - Bi-Annual Data (from 2007 onwards) (Eurostat, 2021; <https://appsso.eurostat.ec.europa.eu/nui/show.do?dataset=nrg_pc_204&lang=en>).
15. Metsä Group, Metsä Group homepage (2019). <https://www.metsagroup.com>.
16. K. Huhtala, "Cooperatives in Finland –What they are Like and How they Operate" (2016; <https://www.chrissmithonline.co.uk/files/kari_edinburgh_2_2016.pdf> (link broken)).
17. O. Lehtonen, L. Okkonen, Energy Cost Reduction Creates Additional Socioeconomic Benefits– The Case of Eno Energy Cooperative, Finland. *Energy Policy*. **129**, 352–359 (2019).<https://doi.org/10.1016/j.enpol.2019.02.018>.
18. S. Ruggiero, A. Isakovic, H. Busch, K. Auvinen, F. Faller, "Developing a Joint Perspective on Community Energy: Best Practices and Challenges in the Baltic Sea Region" (Co2mmunity project, Working paper No. 2.3, 2019; <http://co2mmunity.eu/wp-content/uploads/2019/03/Co2mmunity-working-paper-2.3.pdf>).
19. Statistics Finland, Standard Industrial Classification TOL 2008 (Stat.fi, 2008; <https://www.stat.fi/en/luokitukset/toimiala/>).
20. A. Duffy, M. Hand, R. Wiser, Land-Based Wind Energy Cost Trends in Germany, Denmark, Ireland, Norway, Sweden and the United States. *Applied Energy*. **277**, 114777 (2020). <https://doi.org/10.1016/j.apenergy.2020.114777>.
21. D. Bauknecht, S. Funcke, M. Vogel, Is Small Beautiful? A Framework for Assessing Decentralised Electricity Systems. *Renewable and Sustainable Energy Reviews*. **118**, 109543 (2020). <https://doi.org/10.1016/j.rser.2019.109543>.
22. Bundesverband der Vereine und des Ehrenamtes e.V. (Federal Association of Clubs and Volunteers), Vereine in Deutschland page (2022). <https://bundesverband.bvve.de/vereine-in-deutschland/>.
23. L.H. Broska, It’s All about Community: On the Interplay of Social Capital, Social Needs, and Environmental Concern in Sustainable Community Action. *Energy Research & Social Science*. **79**, 102165 (2021). <https://doi.org/10.1016/j.erss.2021.102165>.
24. S. Centgraf, Supporting Civic Engagement in German Energy Cooperatives – Transdisciplinary Research Based on the Reflection of Individual Needs. *Energy Research & Social Science*. **44**, 112 (2018). <https://doi.org/10.1016/j.erss.2018.05.003>.
25. C. Herbes, B. Rilling, L. Holstenkamp, Ready for New Business Models? Human and Social Capital in the Management of Renewable Energy Cooperatives in Germany. *Energy Policy*. **156**, 112417 (2021). <https://doi.org/10.1016/j.enpol.2021.112417>.
26. B. Schmid, T. Meister, B. Klagge, I. Seidl, Energy Cooperatives and Municipalities in Local Energy Governance Arrangements in Switzerland and Germany. *The Journal of Environment and Development*. **29/1** (2019). <https://doi.org/10.1177%2F1070496519886013>.
27. J. Schwabe, The Evolution of Cooperative Electric Carsharing in Germany and the Role of Intermediaries. *Environmental Innovation and Societal Transitions*. **37** (2020). <https://doi.org/10.1016/j.eist.2020.08.007>.
28. D. Süsser, M. Döring, B.M.W. Ratter, Harvesting Energy: Place and Local Entrepreneurship in Community-Based Renewable Energy Transition. *Energy Policy.* **101**, 332 (2017). <https://doi.org/10.1016/j.enpol.2016.10.018>.
29. A. Wierling, J.P. Zeiss, V. Lupi, C. Candelise, A. Sciullo, V.J. Schwanitz, The Contribution of Energy Communities to the Upscaling of Photovoltaics in Germany and Italy. *Energies*. **14/8**, 2258 (2021). <https://doi.org/10.3390/en1408225>.
30. Core Energy Market Data Registry, (nd). [https://www.marktstammdatenregister.de](https://www.marktstammdatenregister.de/MaStR/Einheit/Einheiten/ErweiterteOeffentlicheEinheitenuebersicht).
31. Cooperatives UK, (2022). <https://www.uk.coop/>.
32. Mutual Public Register, Financial Conduct Authority (2021). <https://mutuals.fca.org.uk/>.
33. M. Tsagkari, How Greece Undermined the Idea of Renewable Energy Communities: An Overview of the Relevant Legislation. *Law Environment and Development Journal (LEAD)*. **17** (2020). <https://www.researchgate.net/publication/346096106_How_Greece_Undermined_the_Idea_of_Renewable_Energy_Communities_An_Overview_of_the_Relevant_Legislation>.
34. I. Douvitsa, The New Law on Energy Communities in Greece. *Cooperativismo e Economía Social*. **40** (2018). <https://doi.org/10.35869/ces.v0i40.1385>.
35. D. Frieden, A. Tuerk, "Deliverable 2.3: Regulatory Frameworks for Energy Communities in the Pilot Site Countries Croatia, Spain, Greece, Portugal and Slovenia, Shaping EU Framework Transposition and Project Implementation" (Compile Project, 2020; <https://www.researchgate.net/publication/346652927_Deliverable_23_Regulatory_frameworks_for_energy_communities_in_the_pilot_site_countries_Croatia_Spain_Greece_Portugal_and_Slovenia_Shaping_EU_framework_transposition_and_project_implementation>).
36. A. Vasilakis,​ C. Vrettos, K. Dimitris, A. Kontolati, G. Koukoufikis, M. Margosi, F. Palaiogiannis, "Mapping of Energy Communities in Greece. HP1AB-00256, Deliverable 1.1" (Green Peace, 2020; <https://www.greenpeace.org/static/planet4-greece-stateless/184045bd-mapping_of_energy_communities_v1.2.pdf>).
37. I. Capellán-Pérez, N. Johanisova, J. Young, C. Kunze, Is Community Energy Really Non-Existent in Post-Socialist Europe? Examining Recent Trends in 16 Countries. *Energy Research & Social Science*. **61**, 101348 (2020). <https://doi.org/10.1016/j.erss.2019.101348>.
38. European Commission. "Commission Staff Working Document, Assessment of the Final National Energy and Climate Plan of Hungary. SWD (2020) 916 Final" (European Commission, 2020; <https://ec.europa.eu/energy/sites/default/files/documents/staff_working_document_assessment_necp_hungary_en.pdf>).
39. International Energy Agency (IEA), "National Survey Report of PV Power Applications in Italy 2020" (IEA Photovoltaic Power Systems Programme, 2020; <https://iea-pvps.org/wp-content/uploads/2021/11/NSR_Italy_2020.pdf>).
40. The Irish Co-operative Organization Society (ICOS), Map of Co-operatives (2021). <http://icos.ie/find-your-co-op/>.
41. Sustainable Energy Authority of Ireland (SEAI), Energy Communities (2022). <https://www.seai.ie/community-energy/sustainable-energy-communities/>.
42. Community Power, Ireland (2021). <https://www.communitypower.eu/en/ireland.html>.
43. Sustainable Energy Authority of Ireland (SEAI), Search our Sustainable Energy Community Network (2022). <https://www.seai.ie/community-energy/sustainable-energy-communities/sec-map/index.xml>.
44. Electric Energy Online, More than 500 Communities are Now Part of SEAI's Sustainable Energy Community Network (2021). <https://electricenergyonline.com/article/energy/category/climate-change/82/889075/more-than-500-communities-are-now-part-of-seai-s-sustainable-energy-community-network.html>.
45. European Commission, "Commission Staff Working Document, Assessment of the Final National Energy and Climate Plan of Ireland. SWD (2020) 906 Final" (European Commission, 2020; <https://ec.europa.eu/energy/sites/default/files/documents/staff_working_document_assessment_necp_ireland_en.pdf>).
46. C. Candelise, G. Ruggieri, Status and Evolution of the Community Energy Sector in Italy. *Energies*. **13/8**, 1888 (2020). <https://doi.org/10.3390/en13081888>.
47. A. Grignani, M. Gozzellino, A. Sciullo, D. Padovan, Community Cooperative: A New Legal Form for Enhancing Social Capital for the Development of Renewable Energy Communities in Italy. *Energies*. **14/21**, 7029 (2021). <https://doi.org/10.3390/en14217029>.
48. M.L.D. Silvestre, M.G. Ippolito, E.R. Sanseverino, G. Sciumè, A. Vasile, Energy Self-Consumers and Renewable Energy Communities in Italy: New Actors of the Electric Power Systems. *Renewable & Sustainable Energy Reviews*. **151**, 111565 (2021). <https://doi.org/10.1016/j.rser.2021.111565>.
49. F. Spinicci, "La Cooperative di Utenza in Italia: Casi di Studio" (European Research Institute on Cooperative and Social Enterprises (Euricse), 2011; <https://www.euricse.eu/wp-content/uploads/2015/03/1323957261_n1894.pdf>).
50. Co2mmunity, "Co2mmunity, Co-Producing and Co-Financing Community Energy Projects, Co2mmunity RENCOPs Summary Booklet, V1.0 (Co2mmunity, 2020; <https://co2mmunity.eu/wp-content/uploads/2020/10/2020_09_30_RENCOP_Booklet.pdf>).
51. Community Power, Latvia (nd). <https://www.communitypower.eu/en/latvia.html>.
52. European Commission, "Commission Staff Working Document, Assessment of the Final National Energy and Climate Plan of Latvia. SWD (2020) 913 Final" (European Commission, 2020; <https://ec.europa.eu/energy/sites/default/files/documents/staff_working_document_assessment_necp_latvia_en.pdf>).
53. E. Laes, K. Anfinson, M. Krug, V. Gatta, E. Meynaerts, E. De Luca, R. Cotroneo, M. Caliano, G. Klāvs, I. Kudrenickis, S. Aakre, H. Sælen, K. Standal, P. Nowakowski, R. Wnuk, I. Azevado, P. Maleki, Assessment Report of Potentials for RES Community Energy in the Target Regions, Deliverable 2.2 (COME RES Project, 2021; <https://come-res.eu/fileadmin/user_upload/Resources/Deliverables/Del_2.2_Assessment_Report_of_Potential.pdf>).
54. European Commission, "Commission Staff Working Document, Assessment of the Final National Energy and Climate Plan of Lithuania. SWD (2020) 914 Final" (European Commission, 2020; <https://ec.europa.eu/energy/sites/default/files/documents/staff_working_document_assessment_necp_lithuania_en.pdf>).
55. Bridge Project, "Energy Communities in the EU - Task Force Energy Communities. Bridge Project, Report D3.12.d" (Bridge Project, 2019; <https://www.h2020-bridge.eu/wp-content/uploads/2020/01/D3.12.d_BRIDGE_Energy-Communities-in-the-EU-2.pdf>).
56. C. Quintana, The Power of Cooperation, Cooperatives Europe Key Figures 2015" (Cooperatives Europe, 2016; <https://coopseurope.coop/sites/default/files/The%20power%20of%20Cooperation%20-%20Cooperatives%20Europe%20key%20statistics%202015.pdf>).
57. European Commission, "Commission Staff Working Document, Assessment of the Final National Energy and Climate Plan of Malta. SWD (2020) 917 Final" (European Commission, 2020; <https://ec.europa.eu/energy/sites/default/files/documents/staff_working_document_assessment_necp_malta_en.pdf>).
58. Malta Co-operative Federation, Co-operative Federation (2021). <https://maltacooperativefederation.coop/>.
59. J. Hufen, J. Koppenjan, Local Renewable Energy Cooperatives: Revolution in Disguise? *Energy, Sustainability and Society*. **5/1** (2015). <https://doi.org/10.1186/s13705-015-0046-8>.
60. F. Boon, C. Dieperink, Local Civil Society Based Renewable Energy Organizations in the Netherlands: Exploring the Factors that Stimulate their Emergence and Development. *Energy policy*. **69**, 297–307 (2014).<https://doi.org/10.1016/J.ENPOL.2014.01.046>.
61. Bedrijvenmonitor, Dutch Business Register (nd). <https://bedrijvenmonitor.info/>.
62. Energie Sammen, Leden en supporters (nd). <https://energiesamen.nu/pagina/1/jouw-belangenvereniging/35/leden-en-supporters>.
63. Esri Nederland, Windturbines (ArcGIS map containing information on wind turbines) (nd). <https://www.arcgis.com/home/item.html?id=4d2e288f6d26406aadba730414faf7a0>.
64. Hier Opgewekt, Lokale Energie Monitor 2020, Excel dataset (2021). <https://www.hieropgewekt.nl/lokale-energie-monitor>.
65. KVK Handelsregister Open Data Set (nd). <https://www.kvk.nl/producten-bestellen/koppeling-handelsregister/kvk-handelsregister-open-data-set/>.
66. D. Lugo-Laguna, A. Arcos-Vargas, F. Nuñez-Hernandez, A European Assessment of the Solar Energy Cost: Key Factors and Optimal Technology. *Sustainability*. **13**, 3238 (2021). <https://doi.org/10.3390/su13063238>.
67. Norges Vassdrag og Energi Direktorat (NVE), "Descriptive Study of Local Energy Communities. Thema Consulting Group, Thema Report 2018-20" (NVE, 2018; <https://thema.no/wp-content/uploads/THEMA-Reort-2018-20-Local-Energy-Communities-Report-Final.pdf>).
68. Krajowy Ośrodek Wsparcia Rolnictwa (KOWR), The National Support Centre for Agriculture (KOWR, 2021; [https://www.kowr.gov.pl/uploads/pliki/DI/Spóldzielnie%20enrgetyczne/Rejestr%20spółdzielni%20energetycznych/Wykaz%20spółdzielni%20energetycznych_2021.05.11.pdf](https://www.kowr.gov.pl/uploads/pliki/DI/Sp%C3%B3ldzielnie%20enrgetyczne/Rejestr%20sp%C3%B3%C5%82dzielni%20energetycznych/Wykaz%20sp%C3%B3%C5%82dzielni%20energetycznych_2021.05.11.pdf)).
69. Ministerstwo Aktywów Państwowych (Ministry of State Assets). *Krajowy plan na rzecz energii i klimatu na lata 2021–2030 (National plan for energy and climate for 2021–2030)* (2019).
70. Ministerial registry, (nd). <https://www.gov.pl/web/klimat/polityka-energetyczna-pol>.
71. J. Jazinski, M. Kozakiewicz, M. Soltysik, Determinants of Energy Cooperatives' Development in Rural Areas - Evidence from Poland. *Energies*. **14/2**, 319 (2021). <https://doi.org/10.3390/en14020319>.
72. H. Algarvio, The Role of Local Citizen Energy Communities in the Road to Carbon-Neutral Power Systems: Outcomes from a Case Study in Portugal. *Smart Cities.* **4/2**, 840-863 (2021). <https://www.mdpi.com/2624-6511/4/2/43/htm>.
73. Coopérnico, *Energia verde, sustentabilidade e cidadania* (2021). <https://www.coopernico.org/>.
74. S. Sareen, "Scalar Biases in Solar Photovoltaic Uptake, Socio-Materiality, Regulatory Inertia and Politics" in *Dilemmas of Energy Transitions in the Global South: Balancing Urgency and Justice*, A. Kumar, J. Höffken, A. Pols, Eds. (London: Routledge, 2021). <https://doi.org/10.4324/9780367486457>.
75. S. Cebotari, Against All Odds: Community-Owned Renewable Energy Projects in North-West Romania. *ACME: An International Journal for Critical Geographies*. **18/2**, 513-528 (2019). <https://acme-journal.org/index.php/acme/article/view/1556>.
76. Community Power, Slovakia (nd). <https://www.communitypower.eu/en/slovakia.html>.
77. R. Leal-Arcas, B. Burstein, M. Mattera, Electrifying the Energy Sector: The Case of Slovakia and the Czech Republic. *Kentucky Journal of Equine, Agriculture, & Natural Resources Law* (Queen Mary School of Law, Legal Studies Research Paper no. 329/2020). **13/1**, 1-83 (2020). <https://www.researchgate.net/publication/339200302>.
78. International Renewable Energy Agency (IRENA), "Renewable Power Generation Costs in 2020" (IRENA, Abu Dhabi, 2021; <https://www.irena.org/-/media/Files/IRENA/Agency/Publication/2021/Jun/IRENA_Power_Generation_Costs_2020.pdf>).
79. European Commission, "Commission Staff Working Document, Assessment of the Final National Energy and Climate Plan of Slovakia. National Energy and Climate Plans (NECP). SWD (2020), 924 Final" (European Commission, 2020; <https://ec.europa.eu/energy/sites/default/files/documents/staff_working_document_assessment_necp_slovakia_en.pdf>).
80. J. Palm, K. Reindl, S. Sommer, "Deliverable D3, Description of Polycentric Settings in the Partner Countries. New Clean Energy Communities in a Changing European Energy System (NEWCOMERS) Project, Deliverable D3" (NEWCOMERS, 2020; <https://www.researchgate.net/publication/342336463_New_Clean_Energy_Communities_in_a_Changing_European_Energy_System_NEWCOMERS_Deliverable_D3_1_Description_of_polycentric_settings_in_the_partner_countries/link/5f16cb30a6fdcc9626a437e1/download>).
81. Compile Project, "Installation and Connection of PV in Luče, Slovenia" (COMPILE Project, 2019; <https://www.compile-project.eu/news/installation-and-connection-of-pv-in-luce-slovenia/>).
82. European Commission, "Commission Staff Working Document, Assessment of the Final National Energy and Climate Plan of Slovenia. SWD (2020) 923 Final" (European Commission, 2020; <https://ec.europa.eu/energy/sites/default/files/documents/staff_working_document_assessment_necp_slovenia_en.pdf>).
83. International Energy Agency (IEA), "National Survey Report of PV Power Applications in Spain, 2020" (IEA Photovoltaic Power Systems Programme, 2020; <https://iea-pvps.org/wp-content/uploads/2021/09/NSR_Spain_2020_b.pdf>).
84. I. Heras-Saizarbitoria, L. Sáez, E. Allur, J. Morandeira, The Emergence of Renewable Energy Cooperatives in Spain: A Review. *Renewable and Sustainable Energy Reviews.* **94**, 1036–1043 (2018). <https://doi.org//10.1016/j.rser.2018.06.049>.
85. C. Gallego-Castillo, M. Heleno, M. Victoria, Self-Consumption for Energy Communities in Spain: A Regional Analysis Under the New Legal Framework. *Energy Policy*. **150**, 112144 (2021). <https://doi.org/10.1016/j.enpol.2021.112144>.
86. D. Magnusson, J. Palm, Come Together—The Development of Swedish Energy Communities. *Sustainability*. **11/4**, 1056 (2019). <https://doi.org/10.3390/su11041056>.
87. International Energy Agency (IEA), "National Survey Report of PV Power Applications in Sweden" (IEA, 2020; <https://iea-pvps.org/wp-content/uploads/2021/10/National-Survey-Report-of-PV-Power-Applications-in-Sweden-2020.pdf>).
88. E. Lantz, M. Hand, R. Wiser, "The past and future cost of wind energy" paper presented at the World Renewable Energy Forum 2012, Denver, CO, 13-17 May 2021.
89. T. Wizelius, "Community Wind in Sweden" in *Handbuch Energiewende und Partizipation*, Holstenkamp, L. & Radtke, J., Eds. (Springer VS: Wiesbaden, 1047-1059, 2018). <https://doi.org/10.1007/978-3-658-09416-4_62>.
90. R. Hewitt, N. Bradley, A. Baggio Compagnucci, C. Barlagne, A. Ceglarz, R. Cremades, M. McKeen, I. Otto, B. Slee, Social Innovation in Community Energy in Europe: A Review of the Evidence. *Frontiers in Energy Research*. **7/31** (2019). <https://doi.org/10.3389/fenrg.2019.00031>.
91. International Energy Agency (IEA). *National survey report of PV power applications in Switzerland, 2019*. IEA photovoltaic power systems programme. <https://iea-pvps.org/wp-content/uploads/2020/09/NSR_Switzerland_2019_v2.pdf> (2019).
